# The Effects of Psycho-Emotional and Socio-Economic Support for Tuberculosis Patients on Treatment Adherence and Treatment Outcomes – A Systematic Review and Meta-Analysis

**DOI:** 10.1371/journal.pone.0154095

**Published:** 2016-04-28

**Authors:** Rosa van Hoorn, Ernesto Jaramillo, David Collins, Agnes Gebhard, Susan van den Hof

**Affiliations:** 1 KNCV Tuberculosis Foundation, The Hague, The Netherlands; 2 World Health Organization, Geneva, Switzerland; 3 Management Sciences for Health, Boston, United States of America; 4 Department of Global Health, Academic Medical Center and Amsterdam Institute of Global Health and Development, Amsterdam, The Netherlands; Universidad Nacional de la Plata, ARGENTINA

## Abstract

**Background:**

There is uncertainty about the contribution that social support interventions (SSI) can have in mitigating the personal, social and economic costs of tuberculosis (TB) treatment on patients, and improving treatment outcomes.

**Objective:**

To identify psycho-emotional (PE) and socio-economic (SE) interventions provided to TB patients and to assess the effects of these interventions on treatment adherence and treatment outcomes.

**Search strategy:**

We searched PubMed and Embase from 1 January 1990–15 March 2015 and abstracts of the Union World Conference on Lung Health from 2010–2014 for studies reporting TB treatment adherence and treatment outcomes following SSI.

**Selection criteria:**

Studies measuring the effects of PE or SE interventions on TB treatment adherence, treatment outcomes, and/or financial burden.

**Data collection and analysis:**

Two reviewers independently assessed titles and abstracts for inclusion of articles. One reviewer reviewed full text articles and the reference list of selected studies. A second reviewer double checked all extracted information against the articles.

**Main results:**

Twenty-five studies were included in the qualitative analysis; of which eighteen were included in the meta-analysis. Effects were pooled from 11 Randomized Controlled Trials (RCTs), including 9,655 participants with active TB. Meta-analysis showed that PE support (RR 1.37; CI 1.08–1.73), SE support (RR 1.08; CI 1.03–1.13) and combined PE and SE support (RR 1.17; CI 1.12–1.22) were associated with a significant improvement of successful treatment outcomes. Also PE support, SE support and a combination of these types of support were associated with reductions in unsuccessful treatment outcomes (PE: RR 0.46; CI 0.22–0.96, SE: RR 0.78; CI 0.69–0.88 and Combined PE and SE: RR 0.42; CI 0.23–0.75). Evidence on the effect of PE and SE interventions on treatment adherence were not meta-analysed because the interventions were too heterogeneous to pool. No evidence was found to show whether SE reduced the financial burden for TB patients.

**Discussion and Conclusions:**

Our review and meta-analysis concluded that PE and SE interventions are associated with beneficial effects on TB treatment outcomes. However, the quality of evidence is very low and future well-designed evaluation studies are needed.

## Background

In 2013, 9 million people developed TB and 1.5 million died from this disease [1,2]. TB is the most common cause of death in people with HIV [1]. The treatment duration for TB is long, at least 6 months for drug-susceptible TB and 18–24 months for multidrug-resistant tuberculosis (MDR-TB) that does not respond to the two most effective anti-TB drugs isoniazid and rifampicin. The long treatment, adverse drug reactions during treatment, stigma and financial burden of TB contribute to non-adherence to treatment and unsuccessful treatment outcomes [3–8]. In addition, ensuring patient adherence to treatment through facility-based directly observed therapy (DOT) competes with work related priorities of patients, adding to the financial burden coming from out-of-pocket and indirect costs related to treatment [7,9], even though anti-TB drugs are provided free of charge in most countries [1,10]. The quick improvement of TB symptoms early in treatment also contributes to patients’ stopping treatment prematurely (i.e. loss to follow-up) as competing interests take priority [9,11]. Poor treatment adherence and loss to follow-up increase morbidity, mortality, and the risk of drug resistance development, and can lead to prolonged transmission of TB [12–17].

Adherence to tuberculosis treatment improves the chance of cure and reduces acquisition of drug resistance and ongoing transmission of TB. The use of DOT through a patient-centered approach, which often requires enablers, is recommended to encourage adherence to TB treatment [18,19]. In some settings and circumstances, incentives alone or in addition to enablers are used to motivate patients to adhere to and complete their full course of treatment [9,16,20–22]. Social support through various educational, emotional, and/or material (in-kind or services) interventions are being provided by numerous TB programmes to remove or alleviate barriers to treatment adherence [9,20,23–25], including the financial burden associated with TB illness and its treatment. Despite the fact that different types of social support interventions (SSI) are implemented, countries still struggle to develop systems that are able to provide SSI in an efficient, effective and sustainable way [26]. WHO guidelines for the programmatic management of drug resistant TB and the new End TB Strategy recommend the use of SSI in TB patients, though WHO has not yet systematically assessed the evidence to support such a recommendation [2,19,27]. Hence, a systematic review of relevant literature on the effects of SSI on TB treatment adherence, treatment outcomes, and financial burden will be informative for national and global policy making.

The primary aim of this systematic review was to identify SSI provided to TB and MDR-TB patients and assess the evidence of their effects on treatment adherence, treatment outcomes and financial burden related to TB illness. The secondary aim was to describe the funding sources for and ownership of local organizations in the identified interventions.

## Methods

This review followed standard methods as defined by the Cochrane Handbook for Systematic Reviews of Interventions and the Preferred Reporting Items for Systematic Reviews and Meta-Analysis (PRISMA) guidelines [28,29]. The PRISMA checklist is enclosed in the supporting information (S1 PRISMA Checklist).

### Literature search

In this review we searched for two main categories of SSI, namely PE support and SE support. PE support includes both emotional support through psychological interventions (e.g. counseling by health care workers) and companionship support through provision of help for patients to participate in a social network (e.g. peer counseling for patients and their support network)[19]. We did not consider interventions aimed only at providing improved information or education to TB patients, given the recent systematic review showing a lack of evidence related to TB treatment [17]. In addition, reminder systems were not considered social support interventions [30]. SE support entails delivering services, material goods and/or financial assistance [19,31,32]. Financial assistance was categorized according to Richter et al. [7] as”direct transfers of money, such as cash paid as part of a social security system or a program incentive, transport reimbursements, treatment allowances, and the like that are paid directly to affected individuals”. Indirect assistance was defined as: “indirect transfers through, for example, food packages or vouchers, travel vouchers, and payment of health insurance for individuals, households or families”. Some forms of indirect assistance may also be converted into cash. We included tax exemption under indirect assistance. Enterprise assistance was defined as”training programs or microcredit that aim to assist individuals or families to generate income” [7]. We searched for studies assessing the effects of socio-economic and/or psycho-emotional interventions on treatment adherence and/or treatment outcomes and/or financial burden. The study population consisted of patients initiated on anti-TB treatment, including treatment for MDR-TB.

### Outcome measures

Treatment adherence, treatment outcomes and financial burden were considered as the primary outcome measures. Adherence was calculated as the percentage of prescribed doses actually taken. Treatment outcomes were defined according to WHO definitions, where cure and completed treatment are defined as successful treatment outcomes [1]. Unsuccessful treatment outcomes for active TB treatment included death, treatment failure and loss to follow-up (previously named default). Patients with transfer-out or missing treatment outcomes were excluded from the analysis. As timing of loss to follow-up per individual was not available for studies reporting on treatment outcomes but not treatment adherence, for these studies loss to follow-up was not included in calculation of treatment adherence. Financial burden was reported according to the definitions used in the individual studies. We also extracted information about how the SSI were financed and organized.

### Search strategy

We systematically searched PubMed and Embase for primary articles and reviews reporting on SSI and tuberculosis treatment for human subjects, published from 01 January 1990–15 March 2015, on the grounds that relevant old information would emerge from previous reviews and references lists. We reviewed the reference lists of identified articles, editorials and reviews. Additionally, we hand searched the 2010–2014 abstract books of the Union World Conference on Lung Health to identify recent studies that were not published in the literature yet. Databases were searched using the full text search strategy as described in S1 Web annex. We contacted authors when we were not able to extract required information from the identified publication on the SSI provided and its effects.

### Eligibility criteria

Eligibility of studies was based on predetermined inclusion criteria. Original studies including a description of SSI had to be in place, as well as an evaluation of the association of SSI on treatment adherence, treatment outcome and/or financial burden. This was evaluated either by means of a comparison between outcomes of an intervention group and a group receiving standard support (which could be none or a more limited package), or by means of a comparison of the occurrence of interventions in those with positive and negative outcomes (case-control studies). The search strategy was restricted to certain languages including publications in Dutch, English, French, German, Portuguese, Russian and Spanish. No age restriction was applied. We chose not to exclude studies that did not provide DOT to their patients as there is no hard evidence that DOT in a strict sense (i.e. direct observation of medication ingestion) without the DOT provider supporting the patient through education and counseling improves treatment outcome under programmatic conditions [22,33].

### Data collection and analysis

#### Selection of studies and data extraction

One reviewer conducted the literature search (RH) based on the search strategy developed by all authors. Subsequently, two reviewers (SH, RH) independently examined titles and abstracts retrieved by the search. One reviewer (RH) reviewed full texts and the reference lists of selected articles, and extracted study data, which were then verified by a second reviewer (SH). For data extraction and management, a pre-piloted form was developed to list study characteristics including: study design and study aim, type(s) of patients, type(s) of TB treatment, descriptions of intervention and control group, descriptions of intervention and routine support, coverage of patients that received the intervention, results of the intervention and control group and differences between these groups. Duplicate publications of included studies were taken into account if they provided additional information. When disagreements occurred, a third independent reviewer was consulted and discrepancies were resolved by consensus among the three.

#### Risk of bias and quality of evidence

Risk of bias was assessed separately for Randomized Controlled Trial (RCTs) and Non Randomized Studies (NRS). We used the Newcastle Ottawa Scale for NRS [34] and The Cochrane Collaboration’s Tool for RCTs [35]. Furthermore, an additional assessment was made for Cluster Randomized Trials on recruitment bias, baseline imbalance and loss of clusters [36]. For NRS, we considered <10% of subjects lost as indicative of low risk of bias. The quality of evidence was assessed using the Grading of Recommendations Assessment, Development and Evaluation (GRADE) tool [37–40].

#### Data analysis

All SSI were described, irrespective of inclusion in the meta-analysis. We analyzed the dichotomous outcomes using Risk Ratios (RR) for RCTs and cohort studies, and Odds Ratios (OR) for case-control studies, together with corresponding 95% confidence intervals. Ratios were (re)calculated from the data provided in the publications. Subsequently, the (calculated) intervention effects were combined in the meta-analysis. Studies were assessed on clinical diversity (e.g. differences in patient spectrum, type and dose of treatment) and methodological diversity (e.g. differences in methods: blinding of patients, concealment and randomization). Additionally, (statistical) heterogeneity was examined with the I^2^ test along with the visual assessment of the forest plots [28,41]. An I^2^ of 0–40% was considered as low heterogeneity, 30–60% was defined as moderate heterogeneity, 50–90% substantial heterogeneity and 75–100% as high heterogeneity [42]. Furthermore, the I^2^ was interpreted along with the directions and magnitudes of the different studies observed in the forest plots. A p-value for the Chi^2^ test of ≤0.10 was considered as a cut-off point for statistically significant heterogeneity. In case of statistically significant heterogeneity, sensitivity analysis were performed based on patient type (e.g. MDR-TB or not) and risk of bias (e.g. low vs. high risk of bias)[42]. Funnel plots were created to assess for publication bias. To execute the meta-analysis, a random effects model was used, considering the diversity in participants (e.g., susceptible TB-patients and MDR-patients) and interventions (e.g. self-help groups and counseling). The DerSimonian Laird method is based on the inverse-variance approach [42]. Due to the potential heterogeneity of the interventions (PE support, SE support and combined PE and SE support) also stratified analyses were performed [43]. Stata (STATA/SE 13.1) was used to perform the meta-analysis. To visualize the risk of bias assessment, Review Manager (Review Manager (RevMan) 5.3, The Nordic Cochrane Centre, Copenhagen) was used.

## Results

**Fig 1:**
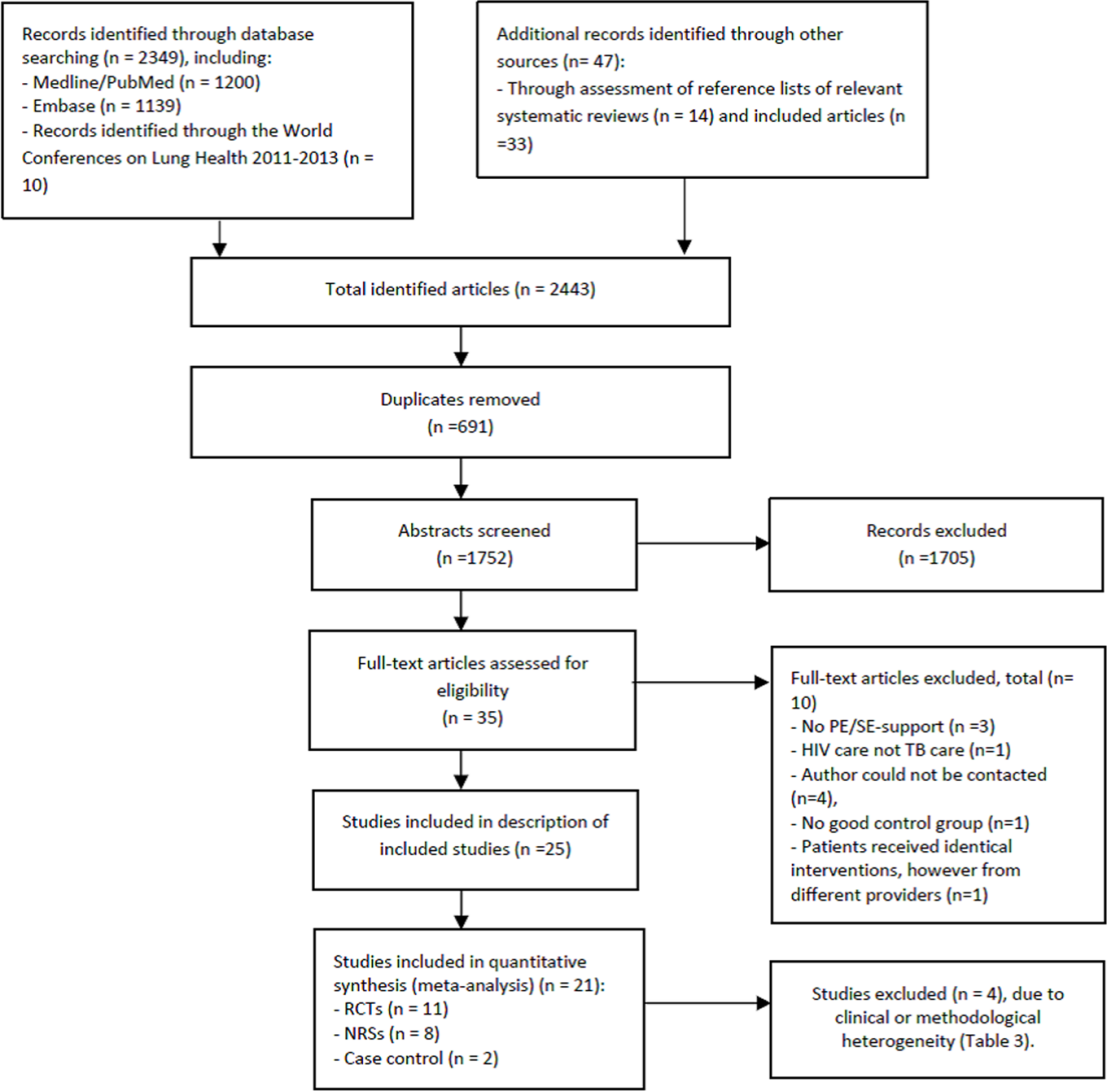
Flow diagram for review and meta-analysis.

In total, we identified 2443 articles. After removal of 694 duplicates, two reviewers screened titles and abstracts of the 1752 citations. Twenty-five articles were eligible for inclusion in the description of included studies (Fig 1).

### Description of included studies

Fourteen NRS and eleven RCTs were included in the description of interventions from 15 different countries. Study populations ranged from 46 to 4,091 participants. Eight studies included both children and adults [44–51]. Three studies explicitly included adults [52–54]. For the other studies the age range was not reported, however mean age was provided frequently [20,55–64]. Most studies were conducted in middle income countries, 9 in upper middle income countries and 7 in lower middle income countries [65]. Six studies were performed in high income countries and the remaining three studies in low income countries. Eleven studies provided SE support only, seven studies provided only PE support, while the remaining seven studies provided a combination of PE and SE support [44,52,56,57,61,66,67] (Table 1). Table 2 includes a comprehensive summary of studies including the frequency of the intervention provided and sustainability of the below described interventions.

**Table 1:** Overview on types of support and inclusion in the quantitative analysis.

	Counseling	Self-help groups	Stigma reduction	Psychotherapy	Involvement of a treatment supporter	Home visits	Other psycho-emotional support	Food supplementation	Other material support	Direct economic support	Indirect economic support	Included in quantitative analysis?
	PSYCHO-EMOTIONAL SUPPORT							SOCIO-ECONOMIC SUPPORT				
*Non-Randomized Studies*												
**Bock et al. 2001 [20]**											X	
**Cantalice Filho 2009 [45]**								X				**X**
**Davidson et al. 2000 [56]**											X	
**Farmer et al. 1991 [57]**						X				X		**X**
**Finlay et al. 2012 [53]**	X											**X**
**Garden et al. 2013 [54]**								X	X			**X**
**Gelmanova et al. 2011 [66]**						X	X		X			
**Jakubowiak et al. 2007 [44]**	X							X	X	X		**X**
**Lu et al. 2013 [48]**										X		**X**
**Macq et al. 2008 [59]**		X	X		X	X						**X**
**Soares et al. 2013 [68]**						X		X	X			
**Sripad et al. 2014 [62]**										X		**X**
**Wei et al. 2012 [63]**										X		
**Zou et al. 2013 [64]**										X		**X**
***Randomized Controlled Trials***												
**Alvarez et al. 2003 [50]**		X										**X**
**Baral et al .2014 [52]**	X									X		**X**
**Drabo et al. 2009 [67]**	X					X		X				**X**
**Jahnavi & Sudha 2010 [58]**								X				**X**
**Janmeja et al. 2005 [55]**				X								**X**
**Liefooghe et al. 1999 [46]**	X											**X**
**Lutge et al. 2013 [47]**											X	**X**
**Martins et al. 2009 [60]**								X				**X**
**Morisky et al. 1990 [61]**	X										X	**X**
**Sudarsanam et al. 2011 [49]**								X				**X**
**Thiam et al. 2007 [51]**	X				X	X						**X**

**Table 2:** Summary table for all studies included in the qualitative analysis.

Study	Country income*	Study type	Enrolment period	N*	Population	Comparison	Intervention(s)	Funding source and organization	Outcome(s)	Other
Alvarez Gordillo et al. 2003 [50]	Chiapas, Mexico,UMIC.	Parallel cluster-randomized study (23 intervention and 25 control health centers).	Febr 2001- Jan 2002.	87 (I: 44, C: 43).	Smear positive pulmonary TB patients 15–89 years, 51% male. Patients with documented resistance were excluded.	Self-help groups vs no support.	Self-help groups. Monthly meetings under coordination of doctors from the specific health unit where the patients received treatment.	Funded by the System Research Benito Juárez, System SEP / CONACYT of Oaxaca, Mexico.	Adherence, defined as Minimal 75% of prescribed dosages taken; treatment completion was defined as 100% of the dosages taken; cure according to the WHO definitions.	Patients could choose the number of meetings and the topics discussed. The health personnel (staff doctors, nurses and social health workers) were trained; they had 6 multidisciplinary workshop days in total. Topics discussed: health, social- economic and cultural aspects of tuberculosis. Theory and practice of diagnosis and treatment of TB and formation of self-help groups.
Baral et al. 2014 [52]	Kathmandu Valley, Nepal,LIC.	Parallel cluster-randomized study.	Jan-Dec 2008.	156 (I1: 33, I2; 42, C: 81).	MDR-TB patients. 83% 21–60 years; 65% male.	1) Counseling only 2) counseling and financial support vs 3) usual care. 7 DOTS plus centers (3:2:2).	1) Counselling on individual level and in small groups, every 2–3 weeks. Or 2) counselling on individual level and in small groups, every 2–3 weeks and US$ 28 per month meant to cover local transport, food and rental costs, but free to use as they chose.	Funded by UK Aid from DFID. Patients receiving financial support were given Nepali Rupees (NRs) 2000 (US$ 28) per month.	Cure, as internationally defined (treatment success).	The intervention was designed after exploratory qualitative study. No adequate sample size calculation (not taking into account clustering), and sample size was smaller than anticipated (partially compensated by including larger number of control patients).
Bock et al. 2001 [20]	Fulton County, Georgia, USA,HIC.	Historically controlled study.	I: Nov 1996—Oct 1997; C: April 1995 -March 1996.	107 (I: 55, C: 52).	TB patients who demonstrated non-adherence by missing at least 25% of DOT doses over a 4-week period. Mean age: 36–38 years; 58% male; HIV infected 34%; alcohol or injection or non-injection drug abusers 56%. Patients, who died, transferred out, lost or uncooperative, were excluded.	Incentive program vs historical controls in the same county. =, who would have been eligible for the incentive under the incentive program.	A coupon redeemable for five dollars in merchandise at a regional chain of grocery stores was given to the patient (or parent/guardian) at each DOT and physician appointment after enrolment. Frequency is unknown.	Partial funding was provided by the Georgia Chapter of the American Lung Association. The cost of incentives for 55 patients was approximately US$ 10.000, less than the cost of treating 1 TB case.	Treatment completion, not defined.	.
Cantalice Filho 2009 [45]	Duque de Caxias, Brazil, UMIC.	Historically controlled study	I:2004 –Jul 2006; C: Sept 2001 –Dec 2003	142 (I: 74, C: 68)	TB patients > 15 years old with confirmed TB. Mean age: 37 years; 59% male; 20% patients with a history of TB; 2% HIV positive.	Treatment and provision of food baskets vs treatment only. Historical controls.	Provision of food baskets on a monthly basis (non-perishable food, the content of the food baskets was not further described).	Funding source is not reported.	Cure, loss-to-follow up, failure and death are not defined.	.
Davidson et al. 2000 [56]	New York City, United States of America, HIC.	Case-control study.	Oct 1992—March 1996.	365 (Cases: 147, controls: 218).	TB patients. Mean age: 40 years; 75% male. 84% were currently unemployed, 74% had no income at the time of the study. 58% was in prison in the past year.	Adherent (attending 80% of the prescribed visits) vs non-adherent patients. Comparison within the same time range. From 6 DOT programs from different city-districts.	10 subway tokens (cash value 15 US$) for attendance at all scheduled appointments each week throughout the course of treatment. Later it changed to 20 tokens a month (cash value 30 US$) at the end of each of the first 2 months and a bonus of 40 tokens at the end of the 3rd month. The 3-month cycles were repeated until treatment ended.	Funded by grants from the Aaron Diamond Foundation and the New York State Department of Health.	Adherence, defined according to a 1990 USPHS report that has been widely cited as a standard for TB treatment. Patients were considered adherent if they attended 80% of their prescribed visits every month of their treatment during the study period.	Not clear what the coverage of support was in the adherent and non-adherent group.
Drabo et al. 2009 [67]	Burkina Faso, LIC.	Parallel cluster-randomized study	Oct 2005-Dec 2007.	333 (I: 178, C: 155).	Smear positive TB patients, further characteristics unknown.	3 intervention vs 3 control districts.	Community groups were raised. Material (food), home visits and psychosocial support was provided to patients. Support was partially provided when needed and educational information was given to the community.	The organizational costs for support committees were included in the annual budgets of respective health districts.	Loss to follow-up, cure and death are not defined.	The community group included 14 people. 2–3 traditional healers, 2 former TB patients, 1–2 community health care workers, 3–4 religious leaders, 2–3 people from community associations and 2 nurses.
Farmer et al. 1991 [57]	Haiti’s central plateau, Haiti, LIC.	Non-randomized controlled study.	Febr 1989—June 1990.	60 (I: 30, C: 30).	(Extra) pulmonary TB (mostly rural) patients. Mean age: 45 years; 33% male; 5% HIV infected patients.	Intervention vs free usual medical care, comparison within the same time frame. Two districts geographically distinct, but are contiguous to each other.	Daily home visits during first month and-, a monthly reminder for clinic visits by the community health worker, and no-show home visits by clinic staff, for food supplements 30 US$ per month for the first 3 months and 5 US$ for travel expenses per month.	Funding source is not reported., however, support was organized by 'Proje Veye Sant'.	Cure: negative sputum smear at the end of treatment (treatment success).	Other support: nutritional supplementation.
Finlay et al. 2012 [53]	8 out of 9 provinces, South Africa, UMIC.	Case-control study.	Jan 1—Dec 31 2002.	1164 (I: 232, C: 932).	TB patients > 18 years old from facility-based national TB registers. HIV rate is unknown. Median age new cases, I: 30 years C: 34 years; median age re-treatment patients, I: 33 C: 39; 58% male.	Patients that were lost to follow-up vs patients that cured, completed or failed treatment. Comparison within a similar time range and geographical location.	Given adequate counselling or information.	Funding source is not reported.	Loss to follow-up is defined as interrupting treatment for two or more consecutive months during treatment.	Also information on TB treatment was measured. Sample selection was conducted by multistage sampling of urban and rural sub-samples.
Garden et al. 2012 [54]	Saint Petersburg, Russia, HIC.	Non-randomized controlled study.	I: 2001–2004, C: 1998–1999.	518 (I:142, C:376).	Homeless TB patients. Age range 23–70. 94% male; 77% has been treated previously for TB; 45% was registered as alcoholics and for 38% no information on this topic was available.	Intervention vs historical controls (no DOT was provided to the controls).	Food incentives, and assistance in providing documentation for health care access and social security	Two Swedish governmental organizations (Swedish East Europe Committee (SEEC) and the Swedish International Development Cooperation Agency (SIDA): Stockholm Sweden).	Loss to follow-up is defined as: when not turning up at the dispensary during three consecutive days. Completion: not interrupting treatment.	.
Gelmanova et al. 2011 [66]	Tomsk City, metropolitan region, Russian Federation, HIC.	Case series (uncontrolled longitudinal study).	17 Dec 2006–30 Nov 2008.	46.	TB patients that participated in at least one intervention to improve adherence before referral to the Sputnik program.68% aged < 38 years; 76% male, 79% was unemployed, 83% had chronic alcoholism, and 72% had MDR-TB.	Before and after the referral to Sputnik’s program. Participants came from all over the Tomsk City region.	More attention and care by health staff, psychological and social assistance (e.g. clothing and assistance with procuring documentation required to access state social service).	Funding source is unknown. The 'Sputnik' program was implemented as a joint program by the Tomsk Oblast Tuberculosis Services (TOTBS) and Partners in Health (PIH).	Adherence: the proportion of doses taken over the total prescribed. Loss to follow-up if they missed all doses for 2 consecutive months. Cure, death and failure according to international consensus definitions	Sputnik’ has a high nurse to patient ratio (2:15), more staff time per patient, provision of cellular telephones to nursing staff (which increases flexibility and easier access to specialists and expanded social and psychological support). Program nurses had training on how to care for patients facing myriad bio-social challenges).
Jahnavi& Sudha 2010 [58]	16, villages in India, LMIC	Randomized controlled study.	Aug–Dec 2005	100 (I: 50, C: 50)	TB cases, culture or sputum positive; BMI < 20. 89% aged 18–65 years old; mean age 37; 74% male; Patient with HIV, DM or other severe underlying diseases were excluded.	Food supplementation and dietary plan vs only general advice and instructions to increase food intake.	Advice on dietary intake with locally available foods was provided to the patient, to meet the target intake of 35 kcal /day/kg body weight. Every day, the patients also received sweet balls made from wheat flour, caramel, groundnuts and vegetable ghee (6 grams protein and 600 kcal of energy), and 100 grams of sprouted grams and nuts for vitamins and minerals), to be consumed in presence of community worker.	Funded by the Padova University, Italy.	Cure: when initially smear-positive who completed treatment had negative smear results on at least two occasions. Completed: When an initially smear-negative patient received the full course of treatment. Death: patients who died during the course of the treatment regardless of the cause.	The community worker ensured that these supplements were collected and distributed to the patients, and consumed.
Jakubowiak et al. 2007 [44]	Six different regions, Russian Federation, HIC.	Case-control study.	March-Sept 2003.	1527 (I: 1444, C: 84)	New pulmonary smear positive and smear-negative TB patients 16–86 years old. Mean age: 43 years; 73% male; 37% was unemployed; 13% imprisonment history; 24% alcohol abuse.	Success vs default, measured in the same time range, from six different regions.	Varying daily to monthly social and economic support (cost 5–30 US$ per package provided): protein food parcels, food supplementation, hot meal, hygiene kits, clothing and/or footwear, newspapers, board games, reimbursement of travel, legal support, household goods on treatment completion. Psychological support (counselling).	Funded by the WHO, IFRC and local authorities. Now already 20 regions are implementing joint social support programs to motivate patients to adhere to treatment.	Treatment success and loss to follow up are according to the WHO definitions.	Social support was organized and implemented by regional TB services, social welfare services, and the local International Federation of the Red Cross and Crescent Societies (IFRC).The support differed intensely per region. 43.3% of the success group did not received social support. 12.1% of the lost to follow-up group received social support
Janmeja et al. 2005 [55]	Chandigarh, India, LMIC.	Non-randomized controlled study.	2001	200 (I: 100, C: 100)	Confirmed new adult cases of pulmonary and extra pulmonary TB patients. Mean age approximately 31 years; 75% male; 38% illiterate.	NTP program + intervention vs usual NTP program care (routine motivation and education). Measured in the same time range and at the same location.	Psychotherapy (8 sessions combined with drug-collection visits), biweekly during the first two months, then monthly.	Funding source is not reported.	Successful treatment: cure and completed. Cured: 6 months of treatment and negative sputum smear at the end of treatment. Completed: negative sputum smear at 2–6 months, without sputum results at completion. Treatment failure: positive sputum smear or culture at 5 months. Loss to follow-up: stopped taking treatment for 2 months or more.	The themes for psychotherapy sessions were structured according to the conceptual understanding of an individual patient obtained from pretreatment psychological assessment. Costs: 12 US$ per patient.
Liefooghe et al. 1999 [46]	Sialkot, Pakistan, LMIC	Randomized controlled trial.	1 Jan—30 Nov 1995	1019 (I: 504, C: 515)	Adult TB patients, age: 15–45+ years; 42% male; 81% new cases; 40% had a low income job.	Intervention vs. usual explanations and treatment by medical staff. Measurements at one hospital.	Counseling. Patients received individual counseling each time they attended for follow-up assessment, and admitted patients received weekly counselling in the tuberculosis ward. Counseling was combined with health education.	Funded by the Vlaamse Interuniversitaire Raad, the Belgian co-operation and the Damien Foundation. The intervention was conceived within the framework of Bandura's social-cognitive learning theory.	Adherence: drug collection at the drug s at the scheduled appointments. Loss to follow-up: no drug collection for 2 months or more.	The social counsellors had several tasks: verify correct understanding of drug intake, to increase the patients’ motivation, anticipate problems and/or critical moments, to activate a social network and involve family members and to act ombudsperson between the hospital/paramedical team and the patient. Two male and two female para-medics received a 2-week training course in counselling. They belonged to the same socio-economic background as the majority of the patients, and were fluent in the different local vernaculars.
Lu et al. 2013 [48]	Shanghai, China, UMIC.	Controlled before-and-after study.	Baseline 2006 and Intervention 2010	1935 (I: 2006: 961, 2010: 734, C: 2006: 281, 2010: 229)	Migrant active TB cases; 59% male, 64% aged 15–34; 86% new cases.	Intervention group vs control group without support in 2006 and 2010. Both groups consisted of 3 districts that have the same geographical characteristics.	Transportation subsidies of US$ 14.63 a month and living allowances of US$ 4.39 a month.	The initial project was made possible through a governmental special financing program (WHO Regional Office for the Western Pacific)	Treatment success: cure (with bacteriologic evidence of success), or completion (without bacteriologic evidence of success).	.
Lutge et al. 2013 [47]	KwaZulu-Natal, South Africa, UMIC.	Randomized controlled trial.	July 2009—March 2010	4091 (I: 2107, C: 1984)	Adults and children diagnosed with pulmonary, drug-sensitive TB, mean age: 31 years; 52% male; 49% HIV positive patients; 56% unemployed.	Incentive treatment vs usual care. 20 public sector clinics were enrolled in rural and urban districts (10:10)	15 US$ voucher was offered to patients every month on collection of their treatment, to a maximum of eight months. Vouchers were redeemed at local shops	Governmental funding.	Successful treatment, the sum of those patients cured and completing treatment. Loss to follow-up and failure was a secondary outcome, however not defined.	In many cases nurses withheld vouchers from eligible patients whom they felt were relatively better off financially.
Macq et al. 2008 [59]	9 rural municipalities, Nicaragua, LMIC.	Non-randomized controlled study.	Diagnosed between March 2004 and July 2005	286 (I: 122, C: 146)	New AFB positive TB patients. Average age: 35 years; 73% male; 49% without declared income.	5 intervention municipalities vs 4 control municipalities (these are the municipalities were the intervention was not effectively implemented).	Strengthening the TB patients through TB clubs taking the form of self-help groups. Additionally arranged home visits, reduce stigma and choice of DOT supporter. At least home visits and TB clubs were implemented in de intervention municipalities	TB clubs were chaired by TB patients and appointed an executive board. A local NGO supported this. The project influenced the National policies about the care of TB in government health services. The National TB program of the Nicaraguan Ministry of Health, the administer of the Global Fund (the NGO NICASALUD), the Damian Foundation (Belgian NGO) and a public health school were involved	Treatment success and loss to follow-up (and stigma reduction), no definition(s) available.	The aim was to increase the relationship between health personnel and TB patients and their realities through performing patient centered home visits to support the patient. And also plan social network activities the patient during the treatment. The interventions received full participation of MOH authorities. And TB clubs had been included in the 2005 Global Fund grant for Nicaragua.
Martins et al. 2009 [60]	Dili, Timor-Leste, LMIC.	Randomized controlled trial.	March 2005—Nov 2005	270 (I: 137, C: 133)	Outpatient participants with newly diagnosed pulmonary tuberculosis. Mean age: 33 years; 65% male; 43% unemployed.	Routine care and nutritional support vs routine care and nutritional advice. The moment of measurement differed between the two groups. From 3 community districts, geographically distinct zones.	Food provision. The participants received food every time they attended the clinic. In the intensive phase, each day they were provided with one bowl food. During the continuation phase, patients were given a food parcel containing unprepared food to take home; quantities were for one meal per day.	Funded by Unicef/UNDP/World Bank/WHO Special program for research and training in tropical diseases	Adherence: not defined. Completion: the clearance of acid fast bacilli from the sputum after treatment or the completion of eight months of treatment, or both, including cure.	.
Morisky et al. 1990 [61]	California, United States of America, HIC.	Randomized controlled trial.	Nov 1985—March 1987	88 (I: 43, C: 45)	Subjects receiving preventive therapy and subjects receiving treatment for active TB (divided into two subgroups). Mean age: 35 years; 55% male.	Intervention vs standard clinic treatment including the use of community workers. Interventions and control came from the same 2 districts.	Health education counselling for 5–10 minutes and 10 US$ (in coupons) at every monthly visit and 40 US$ at the end of treatment (in coupons). (An incentive scheme to reward positive health behaviors plus targeted educational counseling session).	Funded by centers for Disease control. Assistance of the project ‘Clerk’, the project health educators and clinical staff	Treatment adherence: 95% of prescribed medicines taken. And the extent to which a person's behavior (in terms of keeping appointments, taking medications, and executing life-style changes) coincides with medical advice. Loss to follow-up was not defined.	When an active case missed a clinic appointment (interventions and controls), clinical personnel contacted that individual by phone or by home visit to reschedule a new appointment. Intervention subjects were questioned about their specific regimen, and any misunderstandings concerning their medical treatment program were clarified.
Soares et al. 2013 [68]	Rio de Janeiro, Brazil, UMIC	Historically controlled study.	Controls: 2001–2003 and intervention: 2003—Jun 2008	2623 (I: 1771, C: 852)	TB cases from an urban slum	Intervention group vs historical control group without support. Similar geographical location. No DOT provided in control group	DOT, establishment of community health care workers (CHWs) who, led by nurses, established a supportive social network, through this activity the team managed useful services such as transport to TB clinics and donation of food baskets. Also, they and carried out educational activities to enhance TB awareness and promoted breakfasts for patients and their families. The CHWs also collected sputum at home, monitored medical appointment attendance, sent contacts for evaluation and made home visits to supervise treatment	Funded by United States Agency for International Development through the Johns Hopkins University and the US National Institutes of Health Fogarty International Center, Bethesda USA	Treatment outcome (and TB notification rates).	Additionally educational activities were supported. The program was an ongoing training program based on regular feedback of the results of the local team and an on-site supervision scheme implemented by the City TB Program staff. The CHW's have contact with the municipal government, which minimizes employee turnover, making the team stable and avoiding the need for constant training. Regimen was intermittent (twice weekly) in continuation phase during intervention.
Sripad et al. 2014 [62]	Four regions, Ecuador, UMIC.	Historically controlled study.	Jan 2010-Aug 2010, and from Aug 2011-Jan 2012	191 (I: 105, C: 86)	DR-TB patients [resistance to at least one FLD] that received in-patient care for three months and then outpatient care. Mean age: 38 years; 73% male; 63% MDR TB.	Intervention group vs historical control group without support. 3 different regions vs whole Ecuador.	All DR-patients received a US$240 bonus after each month of adherence, defined as taking medications on 26 days per month for up to 24months. They can spend their bonuses according to their needs. They planned to spend their money on food, vitamins, rent, transportation, children’s needs and medicine mainly.	The program was covered by governmental funds. Payments were arranged by the Central Bank of Ecuador, the Ministry of Economic and Social Inclusion and the NTP.	Loss to follow-up rate, not defined.	The program is part of the Ecuador's National Tuberculosis Program (NTP) NTP is a branch of the Ministry of Public Health, is a DOTS-based program with its headquarters in Quito
Sudarsanam et al. 2011 [49]	Southern Indian state of Tamil Nadu, India, LMIC	Randomized controlled trial.	Jan 2005 –Nov 2005	97 (I: 48, C: 49)	Newly diagnosed TB patients. Age: >12 years; 61.2% male; 20.6% HIV positive	Supplementation vs non-supplementation group	The supplementation group received a mixture of cereal and lentil. Three servings a day were provided (930 kcal and 31.5 g protein) and an one-a-day multivitamin tablet.	Funded by the Fogarty AIDS International Research and Training Program and the Global Infectious Disease Research Training grant	Cure: pulmonary smear-positive, completed treatment and had negative smear results on two occasions, one of which is at the end of treatment. Completion: Either pulmonary smear positive, completed treatment with negative smears at the end of the intensive phase but none at the end of treatment or pulmonary smear-negative or extra pulmonary and completed treatment. Unsuccessful: failure, death and loss to follow-up	.
Thiam et al. 2007 [51]	Senegal, LMIC.	Randomized controlled trial.	June 2003—May 2004	1522 (I: 778, C: 744)	Newly diagnosed smear positive pulmonary TB. 88% between 15–49 years; 67% male.	Intervention vs usual NTCP care. Geographical locations of the groups differed. Participants from 16 government districts in Senegal (8:8).	Reinforced counseling and communication between health personnel and patients, involving community health workers, choice of DOT supporter and reinforcement activities.	Funded through a special program from the French Ministry of research, called PAL, which was granted in September 2000.	Cure: negative sputum smear at 8 months and on at least 1 previous occasion. Completion: missing smear results but who had finished their treatment regimen. Loss to follow-up: definitely stopped treatment before completion.	The total support was divided into four components: improving counseling and communication between health personnel and patients through appropriate training, decentralizing treatment to remote health posts and involving community health workers, strengthening the DOT strategy by giving patients the opportunity to choose their treatment.
Wei et al. 2012 [63]	Shanghai, China, UMIC.	Controlled before-and-after study.	Baseline: July 2006—Sept 2007, intervention period: Oct 2007 –Dec 2008	183 (I: 90, C: 93)	Migrant pulmonary TB cases. Average age approx. 33 years; 9% male; 13% illiterate or semi-illiterate. 83% employed.	Intervention group vs control group without support. The two district names were anonymous to protect the patient’s identities.	2 US$ per month for transportation for all migrants, and for all poor migrants (after assessment of poverty) a living allowance of 157 US$ was provided (in four installments 47 US$ at the time of diagnosis, 47 US$ at the end of the second month of treatment, 31 US$ at the end of the fourth month of treatment and 31 US$ at the end of the treatment). 78% and 60% of I and C were assessed to live in poverty; 60% of those in I received a living allowance and the transport subsidy.	Funded by the government. The intervention was designed to fit into the routine practices and job descriptions of the health providers from the CDC, TB clinic in the designated hospitals, and CHCs.	Loss to follow-up: the proportion of migrant TB patients who defaulted from treatment. Completion: the proportion of TB patients who have successfully completed treatment among all the migrant TB patients (treatment success). Financial burden: Percentage of total costs.	Incremental cost-effectiveness analysis. In total, this project involved an investment of RMB 52,400, which consisted of RMB 46,000 of financial subsidy and RMB 6,400 of transport incentives. This additional cost prompted an increase of 8% in treatment completion rate in the intervention district as compared to the control district. This suggests that for each percent increase in treatment completion, an additional cost of RMB 6,550 (US$ 1301) was invested in the intervention district. Similarly, this additional cost delivered a reduction of 10% in the default rate in the intervention district compared with the control district, showing that an additional cost of RMB 5,240 (US$825) was needed to reduce each percent in default rates.
Zou et al. 2013 [64]	Shanghai, China, UMIC.	Controlled before-and-after study.	For baseline: July 2006—Sept 2007. For intervention: Oct 2007—Dec 2008	787 (I1: 90, baseline: 143. I2: 173, baseline: 155. C:93, baseline 133)	Rural to urban migrant active TB cases. Average age, I1: 30, I2: 33, C: 35 years; more patients from I1 and I2 came to Shanghai alone (65% and 47% compared to 30%) other characteristics for the whole population are unclear.	Intervention 1 or intervention 2 vs control group without support. Participants came from 3 districts in downtown Shanghai (1:1:1)	1: A living subsidy of US$ 146 was provided to each poor migrant TB patients (after financial assessment) in four instalments. Every migrant also received US$ 1.50 per month as a transportation incentive. 2: All TB patients, regardless of economic status received a living subsidy of US$ 114 (US$ 19 per month over 6 months) and a transportation incentive of 4.4 US$.	Intervention 1 funded by the Communicable Disease Research Consortium (COMDIS) for the UK Aid Program. Intervention 2 was funded by the Global Fund. The COMDIS approach did not require extra investment from the health provider as the Global Fund approach did. The COMDIS approach might achieve better cost savings as it focused on providing financial incentives only to poor migrant TB patients	Treatment success (completion and cure), loss to follow-up and death. Nod definitions available. Financial burden was described as: cost-effectiveness.	For each percent increase in treatment completion, an additional cost of US$ 1301 was invested in the intervention district. For each percent decrease in loss to follow-up additional costs of US$ 825 was needed.

#### Psycho-emotional support

Seven studies provided counseling, exclusively [46,53] or in combination with other PE and or SE interventions [44,51,52,61,67]. The scope of the additional interventions varied from food supplementation [44] combined with home visits [67], direct economic support constituted after an exploratory quality study [52], cash coupons at every monthly visit and at the end of treatment [61], arrangement of a self-chosen treatment supporter [51]. See Table 2 for details.

Furthermore, 2 studies organized self-help groups [50,59], one of these studies along with stigma reduction and home visits [59]. TB clubs were raised in the form of self-help groups in combination with support to reduce stigma and home visits to get insight in the social network of the patients and to plan activities to support the patient [59]. In the second study, the patients could choose the number of meetings and the topics discussed [50]. Another 6 studies arranged home visits together with other interventions [51,57,59,66–68].

#### Socio-economic support

Eight studies provided food supplementation consisting of fresh food supplies [58,60], hot meals [44] and/or food packages [44,45,49,54,60,67,68]. Four of them exclusively provided food supplementation [45,49,58,60]. Other studies also provided food supplementation, in combination with direct economic support and/or other material support through provision of e.g. clothing and legal support [44], assistance in providing documentation for health care access and social security [54], or establishing a supportive social network of organizations that could provide support to the local community, such as public day care centers and employment agencies [68]. One study additionally provided PE support [67].

Four studies provided indirect economic support including food and transport vouchers [20,47,56,61]. Coupons varying from 5 to 15 US$ were given when attending each appointment or at drug collection each month. Some studies provided additional coupons varying from 40 to 60 US$ after completion of 3 months of treatment or at the end of treatment [56,61]. Seven studies granted direct economic support, mainly financial support varying from 19 to 240 US$ per month [44,48,52,57,62–64]. Four studies provided direct economic support exclusively [48,62–64]. Other studies only provided economic support for the first three months and 5 US$ per month for travel expenses [57] or arranged reimbursement of travel for an unknown amount of money, combined with food supplementation, other material support and psycho-emotional support [44]. The remaining three studies also combined socio-economic support with psycho-emotional support. No studies on ‘enterprise assistance’ were found. Details on economic support provided per study are retrievable in Table 2.

#### Funding sources and organization

Information on funding sources and involvement of local bodies in the organization of the interventions can be found in Table 2. Seven SSIs were financed through governmental funding or local authorities. Another nine interventions were funded by foreign donor assistance (e.g. WHO, Unicef). Three interventions received combined funding (local and foreign donor assistance). For the remaining five interventions the funding source was unknown.

In total nine studies provided information on the organization of interventions, including six RCTs [46,50–52,55,67] and three NRS [44,59,66]. A study from Russia organized and implemented support by regional TB services and a local international organization[23] and a study from Nicaragua raised TB clubs organized by TB patients, with the help of local non-governmental organizations [59]. Community involvement was integrated into regular patient management in Burkina Faso [44,59,67]. The remaining studies reported very limited information on organizational sustainability.

#### Incentives and enablers

All the RCTs defined their support as incentives. Incentives are rewards for adherence while enablers assist patients to overcome barriers to treatment adherence. Most studies provided support to all TB patients. In studies where only poor patients were supported [64]; it may be that the support in fact was in the form of enablers.

### Risk of bias and quality of evidence

**Fig 2:**
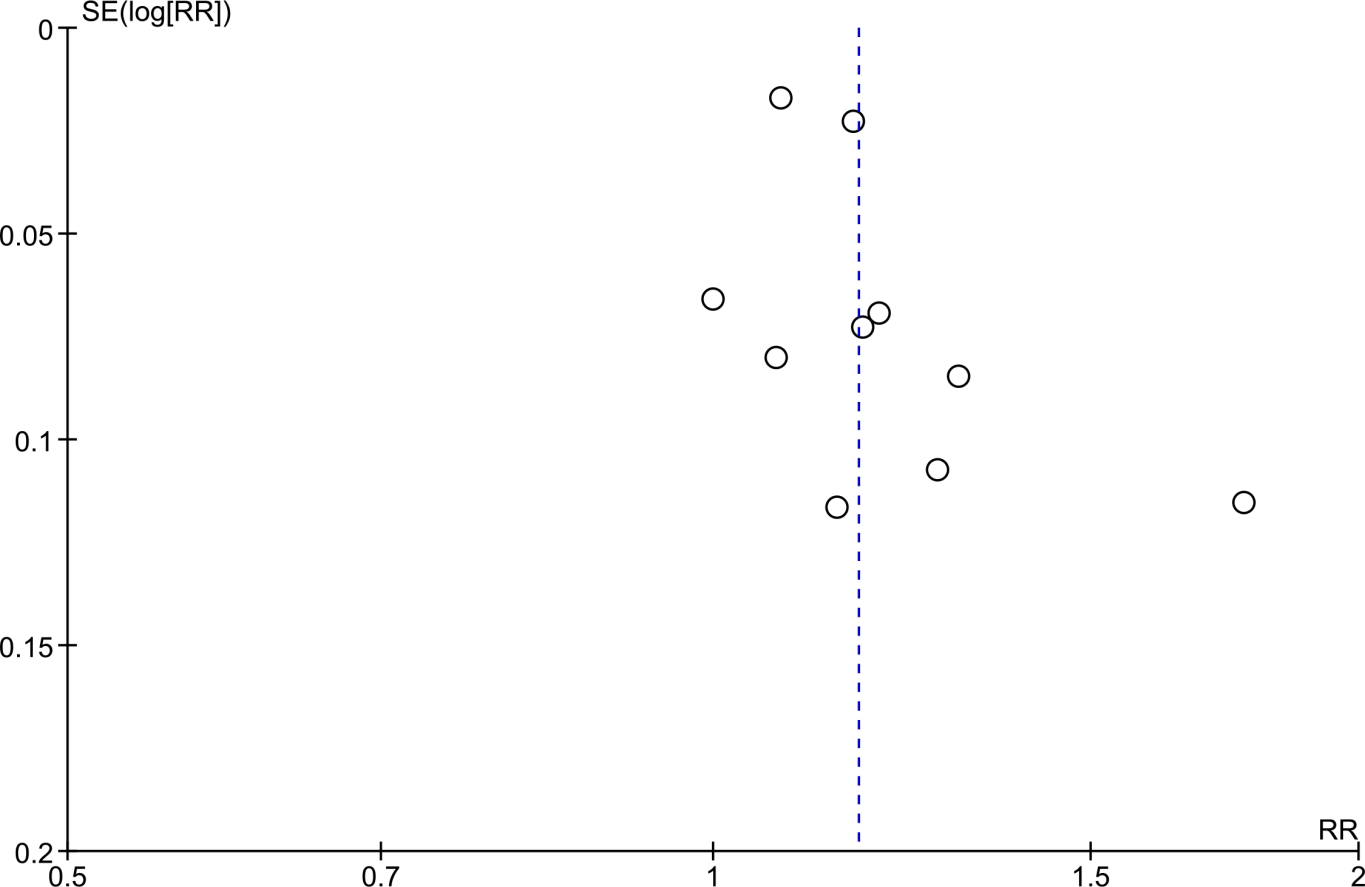
Funnel plot to evaluate publication bias in Randomized Controlled Trials on the effects of social support interventions on treatment outcomes.

Risk of bias was assessed for all included RCTs, including six Cluster Randomized Trials [47,50–52,60,67]. Only five out of eleven RCTs described an adequate randomization approach [50–52,58,60]. For the majority of the studies it was not described whether investigators were blinded to the outcome, and assessment of reporting bias was not possible due to a lack of information. None of the Cluster Randomized Trials assessed baseline imbalances between clusters or took random effects into account in the analysis. Ten NRS were assessed on risk of bias, including eight cohort studies and two case-control studies. Four studies [20,56,63,66] were not included in the meta-analysis and risk of bias assessment; reasons for exclusion are described in Table 3. Only three NRS adjusted for one or more confounders in the analysis [44,48,53]. Five additional studies were not included because of inadequacy of follow-up and/or assessment of outcome measures [44,48,53,62,68]. More information on the risk of bias assesment of the RCTs and NRS can be found in the supportive information S1–S3 Tables. Quality of evidence was assessed for the included RCTs per outcome measure. The quality of evidence for the RCTs was downgraded with one level for risk of bias, two levels on indirectness of studies and one level for limitations in consistency of the results. Hence, the overall quality of evidence of this systematic review is considered to be very low [40,69–74]. The quality of evidence per outcome measure is similar to the overall quality of evidence and retrievable in the summary of findings table (Table 4). No rating up for the overall quality of evidence was possible. Based on the funnel plot for the results of the ten RCTs included in the meta-analysis, it was not possible to determine whether publication bias was present (Fig 2)[28]

**Table 3:** Studies excluded from quantitative analysis.

Study	Type of study	Population	Dot	intervention	Outcome	Effect	Reason(s) for exclusion
Bock 2001 [20]	Historically controlled study	Non-adherent TB patients	Yes	Indirect economic support	Adherence	≤32 weeks OR 5.73 [CI 2.25–14.84] ≤52 weeks 7.29 [2.45-22-73]	Methodological diversity: outcome different than in other studies.
Davidson 2000 [56]	Case-control study	TB patients	Yes	Indirect economic support	Adherence	The odds that a patients with 100% adherence under incentives program will adhere 2.7 (1.01^**100)**^ times as great as person receiving the basic incentive package.	Methodological diversity: not possible to calculate absolute numbers from the effects.
Gelmanova 2011 [66]	Case series	TB patients that participated in at least one intervention to improve adherence before referral to the Sputnik program.	Yes	Home visits, other psychological and other social support	Adherence	Increased from 52.2% [CI 47.5–56.9] to 81.4% [CI 76.8–86.0]	Methodological and clinical diversity: high risk of bias on the ‘selection’ and ‘outcome’ domain (S2 Table). Study population only includes non-adherent patients, which were their own controls.
Wei 2012 [63]	Controlled before–and–after study	(Poor) Migrant TB patients	Unclear	Direct economic support	Treatment success, loss to follow-up and death.	Significant reduction of default rates (11% vs 1%, P = 0.03) in intervention district compared to the control district	This study was part of a bigger study (Zou et al. 2013 [64]), therefore this study was excluded.

**Table 4:** Summary of findings.

Outcomes	Social support intervention(s)	Relative risk (CI)	Number of participants (studies)	Quality of evidence*	Risk of bias	Inconsistency	Imprecision	Indirectness
Treatment success	Social support interventions (overall)	1.17 (1.09–1.25)	6547, 10 studies	Very low	Serious risk of bias	Serious inconsistency, downgraded with one level due to high heterogeneity (I^2^ of 72.8%, P = <0.001).	No serious imprecision, adequate sample size (n = 345).	Very serious indirectness
Treatment success	Psycho-emotional support	1.37 (1.08–1.73)	400, 3 studies	Very low	Serious risk of bias, downgraded with one level for high risk of bias on two domains for one study.	Serious inconsistency, downgraded with one level due to high heterogeneity (I^2^ of 78%, P = 0.011) and large variation in point estimates.	No serious imprecision, adequate sample size (n = 44)	Very serious indirectness, downgraded with two levels. The studies provided different PE interventions (counseling, psychotherapy and self-help groups). One study provided the intervention to a different population (MDR-TB patients). In addition, mostly indirect comparisons are made.
Treatment success	Socio-economic support	1.08 (1.03–1.13)	4324, 4 studies	Very low	Serious risk of bias, downgraded with one level on high risk of bias on one domain in three studies.	No downgrading for inconsistency	No serious imprecision, adequate sample size (n = 748).	Very serious indirectness, downgraded with two levels. Three included studies provided food supplementation; one study provided indirect economic support. In addition, mostly indirect comparisons are made.
Treatment success	Combined support	1.17 (1.12–1.22)	1823, 3 studies	Very low	Serious risk of bias, downgraded with one level for high risk of bias on two domains in one study.	No downgrading for inconsistency	No serious imprecision, adequate sample size (n = 133).	Very serious indirectness, downgraded with two levels. All studies provided counseling and one or more PE and/or SE interventions. One study provided the intervention to a different population (MDR-TB patients). In addition, mostly indirect comparisons are made.
Unsuccessful treatment outcomes	Social support interventions (overall).	0.53 (0.41–0.70)	7301, 10 studies	Very low	Serious risk of bias	Serious inconsistency, downgraded with one level due to high heterogeneity (I^2^ of 80.2%, P = <0.001) and large variation in point estimates.	No serious imprecision, adequate sample size (n = 358)	Very serious indirectness
Unsuccessful treatment outcomes	Psycho-emotional support	0.46 (0.22–0.96)	1419, 4 studies	Very low	Very serious risk of bias, downgraded with two levels for high risk of bias in two studies with high risk of bias on two domains.	Serious inconsistency, downgraded with one level due to high heterogeneity (I^2^ of 85.5%, P = <0.001) and large variation in point estimates.	No serious imprecision, adequate sample size (n = 267).	Very serious indirectness, downgraded with two levels. The studies provided different PE interventions (counseling, psychotherapy and self-help groups). One study provided the intervention to a different population (MDR-TB patients). In addition, mostly indirect comparisons are made.
Unsuccessful treatment outcomes	Socio-economic support	0.78 (0.69–0.88)	3967, 2 studies	Very low	Serious risk of bias, downgraded by one level for high risk of bias on one domain in 2 studies.	Serious inconsistency, downgraded with one level due to large variation in point estimates (RR = 0.2 and 0.78).	No serious imprecision, adequate sample size (n = 1059).	Very serious indirectness, downgraded with two levels. The studies provided different SE interventions (food supplementation and indirect economic support). In addition, mostly indirect comparisons are made.
Unsuccessful treatment outcomes	Combined support	0.42 (0.23–0.75)	1915, 4 studies	Very low	Serious risk of bias, downgraded by one level for high risk of bias on two domains in one study and one study with high risk of bias on one domain.	Serious inconsistency, downgraded with one level due to high heterogeneity (I^2^ of 64.2%, P = 0.039) and large variation in point estimates.	No serious imprecision, adequate sample size (n = 127).	Very serious indirectness, downgraded with two levels. All studies provided counseling and one or more PE and/or SE interventions. One study provided the intervention to a different population (MDR-TB patients). In addition, mostly indirect comparisons are made.

* GRADE Working Group levels of evidence.

### Meta-analysis

**Fig 3:**
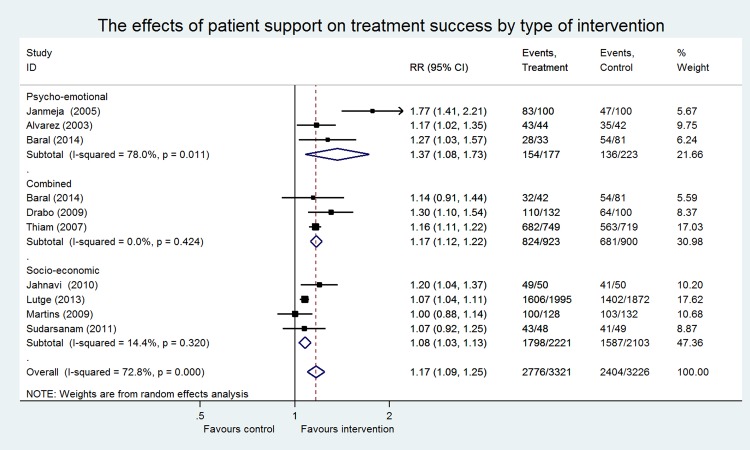
The effects of social support on treatment success by type of intervention in Randomized Controlled Trials.

**Fig 4:**
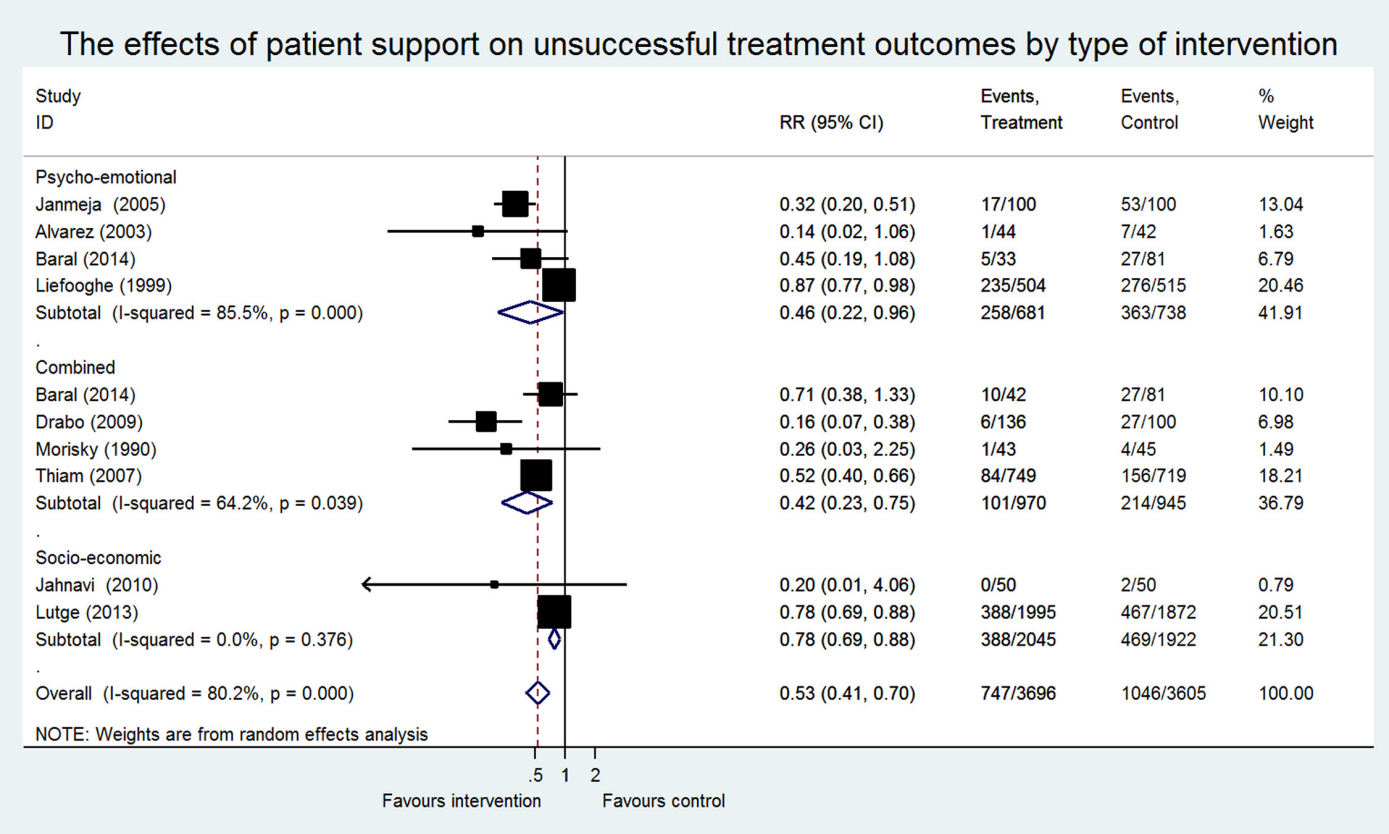
The effects of social support on unsuccessful treatment outcomes by type of intervention in Randomized Controlled Trials.

Eleven RCTs, eight cohort studies, and two case-control studies were included in the meta-analysis, including 17 743 patients (9655 patients participating in RCTs and 8088 patients in NRS). Most data originated from Brazil, China, Russia, Senegal and South Africa. No evidence was found concerning the effect of SSI on financial burden. Only one NRS measured the cost-effectiveness ratio of the provided economic support [64]. Studies assessing the effect of SSI on treatment adherence were too heterogeneous to pool. Meta-analysis of different outcome measures are presented separately (Figs 3 and 4).

#### Treatment outcomes

In total, nine RCTs had treatment success as an outcome measure (Fig 3). The overall effect of these studies showed a significant positive effect (RR 1.17; CI 1.09–1.25), however significant heterogeneity was observed (I^2^ of 72.8%, P = <0.001). Stratified analyses were performed for the different types of interventions. Three studies provided PE support [50,52,55] including counseling, psychotherapy and the organization of self-help groups. A significant pooled effect was found for this intervention (RR 1.37; CI 1.08–1.73). The association between SE support and treatment success was examined by four studies [47,49,58,60] providing food supplementation and economic support. A significant pooled effect was found for this intervention (RR 1.08; CI 1.03–1.13). Combined support was provided by three studies [51,52,67]. Also, a significant pooled effect was found for these interventions on successful treatment outcomes (RR 1.17; CI 1.12–1.22). No significant heterogeneity was observed in two of three stratified analyses (SE: I^2^ of 14%, P = 0.32; combined: I^2^ of 0%, P = 0.42). Studies that provided PE support were substantially heterogenic and the p-value for the Chi^2^ test was significant (I^2^ of 78%, P = 0.01) (Fig 3). A sensitivity analysis was performed on the effect of PE support on treatment success, comparing high vs. low risk of bias studies. Omitting one high risk of bias study removed heterogeneity (I^2^ of 0%, P = 0.53) (data not shown), and did not change effect size (RR 1.20; CI 1.07–1.35) [55]. Sensitivity analysis on MDR-TB patients vs. non-MDR-TB patients did not change the effect size and statistical significance (data not shown).

Nine studies had unsuccessful treatment outcomes as an outcome measure including seven also having treatment success as an outcome measure (Fig 4). An overall significant protective effect was found (RR 0.53; CI 0.41–0.70), however, substantial heterogeneity was observed (I^2^ of 80.2% and P = <0.001). Stratified analyses were performed on the different interventions provided. Four studies investigated the effect of PE support on unsuccessful treatment outcomes, including counseling, psychotherapy and the organization of self-help groups [46,50,52,55]. Two studies examined the effect of SE support, including food supplementation and economic support [47,58] and four studies assessed the effect of combined support [51,52,61,67]. A significant reduction in unsuccessful treatment outcomes was found for all three stratified analyses: PE support (RR 0.46; CI 0.22–0.96), SE support (RR 0.78; CI 0.69–0.88) and a combination of PE and SE support (RR 0.42; CI 0.23–0.75). Heterogeneity was considered to be very low for the studies that provided SE support interventions (I^2^ of 0% and P = 0.37). The studies that provided PE support and combined support were substantially heterogenic (PE: I^2^ of 85%, P = <0.001 and combined: I^2^ of 64% (P = 0.03) (Fig 4). A sensitivity analysis was performed in the PE stratum on the basis of higher risk of bias compared to the other studies [46,55]. Removal of one high-risk of bias study [46] decreased the I^2^ to 0% (P = 0.54) and the effect size changed but remained statistically significant (RR 0.33; CI 0.22–0.50). Omitting both biased studies did not change heterogeneity or the effect size. Sensitivity analysis on risk of bias was not possible in the studies providing a combination of PE and SE support, due to the fact that 3 out of 4 studies were classified as biased studies. Sensitivity analyses on MDR-TB patients vs. non-MDR TB patients did not change the effect size or heterogeneity significantly (data not shown).

#### Treatment adherence

Three RCTs assessed the effect of PE and/or SE on treatment adherence. A PE-intervention study conducted in Mexico showed a significant improvement in treatment adherence (RR 1.20; CI 1.03–1.39). A study from the USA did not show significantly higher levels of adherence in the intervention group compared to the group that received usual care (RR 1.11; CI 0.92–1.33). A third study from Timor-Leste showed no effect for patients that received SE support compared to patients that did not receive this support (RR 1.01; CI .0.85–1.21). Above-described interventions were not pooled as they were too heterogeneous.

#### Financial burden

None of the RCTs examined the effect of PE or SE support on financial burden for TB patients.

#### Non-randomized studies

Due to the fact that the studies’ characteristics were heterogeneous on several levels and at higher risk of bias than the RCTs, we chose not to pool the effects for these studies (S1 and S3 Figs) [28,75]. Seven NRSs reported an effect of social support on successful treatment outcomes. Effects of interventions on successful treatment outcomes (RR) ranged from 1.03 to 2.51 (CI 0.96–2.99). Five of seven NRSs reported significant effect sizes [48,54,57,64,68]. Two studies found no significant effects [45,59]. Furthermore, six NRSs examined the effect of social support on unsuccessful treatment outcomes. Effect sizes varied from RR 0.32–0.96 (CI 0.18–3.49). Five out of six NRSs showed significant beneficial effects [45,54,62,64,68]. Only one study reported a non-significant effect [59]. In addition, two case-control studies investigated the effect of social support on unsuccessfull treatment outcomes. Both studies showed significant beneficial effects (RR 0.51 (CI 037–0.70) and RR 0.10 (CI 0.05–0.20)).

## Discussion

This review found that PE and SE support did improve treatment outcomes across a variety of settings and patient populations, with a tendency towards better outcomes with PE interventions or a combined approach. However, the quality of evidence was classified as “very low” under the GRADE approach. Food supplementation and counselling were commonly included in the package of support. PE, SE and combined interventions improved treatment outcomes; only for interventions including SE support exclusively there was no significant improvement in treatment success. Overall, support interventions were associated with significantly higher treatment success (overall RR 1.08; CI 1.03–1.13) and reductions in unsuccessful treatment outcomes (overall RR 0.53; CI 0.41–0.70). Hardly any studies assessed the effect of interventions on treatment adherence. However, improved treatment adherence is an intermediate goal with the final aim to improve treatment outcomes, which was shown to improve.

A recent systematic review concluded that the economic burden for patients is considered to be high, loss of income is an important indirect cost factor for TB patients, and transport and nutritional supplementation were important direct cost components [8]. A study in Peru evaluated the expenses for MDR-TB patients that received free treatment and found that having MDR-TB was associated with high costs, which was associated with adverse outcomes (population attributable fraction 18–20%) [76]. In line with our review, these two studies suggest that economic support is of great importance for improving treatment outcomes. Some of the findings of this review however differ from those from other SSI-related reviews. A recent review [77] on RCTs assessing the effect of material incentives on TB treatment adherence and completion of TB treatment identified two trials, both included in our review as well [47,60], and neither demonstrated a clear benefit. However, in one trial the incentive was not well received by the patients and in the other trial fidelity to the intervention was low. A review of Sinclair et al. did not find any evidence that food supplementation had a beneficial impact on treatment outcomes [78]. This may be explained by their focus on micronutrient supplementation alone as reflected in their search strategy. In a systematic review about strategies to reduce loss to follow-up in drug-resistant patients, a comprehensive package of interventions (e.g. financial support and food supplementation) was associated with reduced loss to follow-up [79]. Our review included studies focusing on all TB patients, not only those with MDR-TB [79]. As mentioned in the methods section, we did not consider interventions aimed only at providing improved information or education to TB patients, given the recent systematic review showing a lack of its evidence related to TB treatment [17]. Some of the intervention packages included in our review included an information or education component, but it was not possible to delineate the effects of this specific component in our review. We also did not include interventions focusing only on reminder systems, as these are not considered PE or SE support. However, reminder systems can be integrated into SSI programs to enhance its effects since pre-appointment reminder phone calls and letters or home visits did have a small but potentially relevant effect on treatment completion [30].

There were some limitations to our review. Only a limited number of studies were available on the effect of PE/SE support interventions on TB treatment outcomes and very limited evidence on treatment adherence and financial burden. Within the identified studies, we were not able to stratify results by the type of organization and quality of health service delivery due to insufficient information, although it is known that organization and quality of health service delivery influence treatment adherence [9]. Some NRSs only provided support to subgroups of patients including poor patients [64], patients that already received support before referral to the intervention studied [66] and non-adherent patients [20]. This precludes conclusions on the effects of these interventions when provided to all patients. Such patient selection may have led to overestimations in the observed effect of the PE/SE interventions. On the other hand, selecting patients most in need seems prudent and is in practice applied in resource-limited settings. Although the number of studies included in the meta-analysis was small, the optimal size criterion was sufficient both for the overall meta-analysis and stratified analyses as examined by calculation of the sample size for the overall effect and subgroup analyses [72]. We could not examine for a dose response rate across all included studies, as most studies did not include a comprehensive description of interventions. However, one study did show a positive dose-response within their study regarding provision of indirect economic support: among patients in the intervention group who received the voucher at least once, treatment success rates significantly improved [47]. Furthermore, the more frequent the vouchers were received by patients, the higher their probability of treatment success [47]. Plausible heterogeneity was observed and seven out of eleven RCTs had a high risk of bias on one or two domains. However, we did not exclude studies on the basis of heterogeneity only, as this may introduce bias [42].

## Conclusions

This review provides evidence to endorse implementation of SSI in order to improve treatment outcomes. Firstly, PE and combined PE/SE support have a beneficial impact on treatment success. Secondly, SE support and a combination of PE/SE support are associated with reductions in unsuccessful treatment outcomes. No conclusions can be drawn considering the overall effect of PE and/or SE support on treatment adherence and financial burden due to a lack of evidence. Our findings need to be interpreted with caution, as the quality of the evidence included in the meta-analysis is “very low” based on the GRADE approach. In addition, most support included multifaceted types of interventions, so no conclusions can be drawn on the effect of individual interventions. Simultaneously, this might signify that multifaceted types of interventions are needed to improve treatment outcomes. High quality evidence, from well-designed randomized studies in larger sized populations, would provide more certainty on the effects of different PE and SE interventions. Cluster-randomized studies would provide an opportunity to compare differential packages and delineate the importance of specific components. In addition, more systematic data collection on PE and SE as already used by TB programs to monitor implementation and evaluate its effects and qualitative data collection in both studies and program settings to assess which interventions are most appreciated and most feasible to implement on a wide scale, would be useful. Reports should include information on costs and sustainability to provide information on efficiency and scalability.

## Supporting Information

S1 PRISMA Checklist PRISMA checklist. (DOC)

S1 Fig The effects of social support on treatment success in non-randomized cohort studies. (PNG)

S2 Fig The effects of social support on unsuccessful treatment outcomes in non-randomized cohort studies. (PNG)

S3 Fig The effects of social support on unsuccessful treatment outcomes in Case-control studies. (PNG)

S1 Table Risk of bias assessment–Cochrane collaborations tool for randomized controlled trials. (DOCX)

S2 Table Risk of bias assessment–New-castle Ottawa scale for non-randomized studies. (DOCX)

S3 Table Risk of bias assessment–New-castle Ottawa scale for case-control studies. (DOCX)

S1 Web Annex Full text search strategy per database. (DOCX)
