# Supplementary figures and images for: The Effects of Psycho-Emotional and Socio-Economic Support for Tuberculosis Patients on Treatment Adherence and Treatment Outcomes – A Systematic Review and Meta-Analysis
Source: PLoS One. 2016 Apr 28;11(4):e0154095. doi: 10.1371/journal.pone.0154095 (PMC4849661; doi:10.1371/journal.pone.0154095)

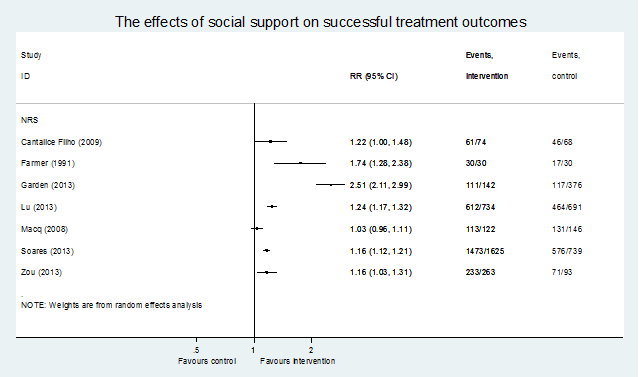

Supplement: S1 Fig — (PNG) [file pone.0154095.s002.png]

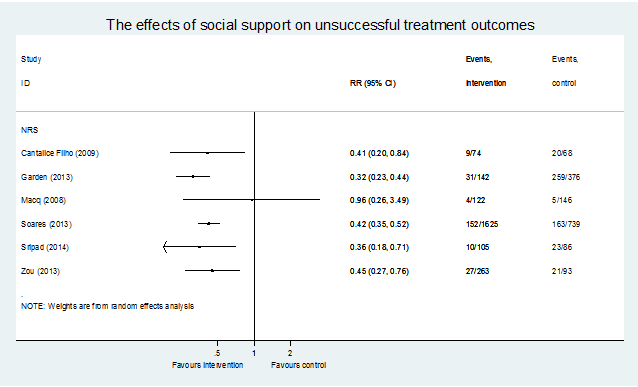

Supplement: S2 Fig — (PNG) [file pone.0154095.s003.png]

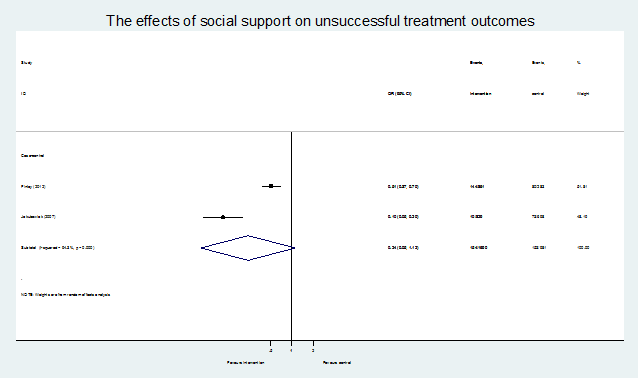

Supplement: S3 Fig — (PNG) [file pone.0154095.s004.png]
