# Supplementary material for: The Effects of Psycho-Emotional and Socio-Economic Support for Tuberculosis Patients on Treatment Adherence and Treatment Outcomes – A Systematic Review and Meta-Analysis
Source: PLoS One. 2016 Apr 28;11(4):e0154095. doi: 10.1371/journal.pone.0154095 (PMC4849661; doi:10.1371/journal.pone.0154095)
Supplement: S1 Table — (DOCX) [file pone.0154095.s005.docx]

# S1 Table. Risk of bias assessment – Cochrane collaborations tool for randomized controlled trials

| Study | Random sequence generation | Allocation concealment | Blinding of participants | Blinding of Personnel | Blinding of outcome assessment | Incomplete data | Selective reporting | Other sources of bias |
| --- | --- | --- | --- | --- | --- | --- | --- | --- |
| *Alvarez [54]* | Parallel cluster randomized trial. Health units were selected at random They were identified with codes on a paper, put into a vase, and then numbers were extracted. | The health units were randomized all at once, no lack of concealment of an allocation sequence. | Not described. Due to the nature of the intervention it is likely that the participants were not blinded. | Not described. Due to the nature of the intervention it is likely that the participants were not blinded. | No information available. | 1 patient (2%) in intervention and 3 patients (7%) in the control group were lost to follow-up. Indication that no clusters were lost. | No information on selective reporting. | Lack of information on recruitment and baseline imbalances. Clustering is not taken into account in the analysis. |
| *Authors judgment* | Sufficient randomization procedure, low risk of bias. | Low risk of bias. | Low risk of bias, the outcome is not likely to be influenced due to lack of blinding. | Low risk of bias, the outcome is not likely to be influenced due to lack of blinding. | No judgment possible, unclear risk of bias | Low risk of bias, due to low loss to follow-up. | Unclear risk of bias. | Unclear risk of bias, low precision due to small number of clusters. |
| *Baral [46]* | Parallel cluster randomized trial. Method of random sequence generation is not described. The 7 DOTS-plus centers were randomly allocated. | The DOTS-plus centers were randomly allocated to the 3 types of care- (2 to counselling, 3 to combined support, and 2 to usual care) – by selecting randomly from the numbers 1 to 7. | Not described. Due to the nature of the intervention it is likely that the participants were not blinded. | Not described. Due to the nature of the intervention it is likely that the participants were not blinded. | No information available. | Loss to follow-up was an outcome measure. No indication that clusters were lost. | No information on selective reporting | Lack of information on recruitment bias and baseline imbalances. Analysis did not take clustering (by DOTS-plus centers) into account and there is a possibility that the groups are not comparable on unrecorded factors. |
| *Authors judgment* | Low risk of bias, 100% coverage of the DOTS-plus centers in the area. However, caution should be taken when generalizing the results. | Low risk of bias, the health units were randomized all at once, no lack of concealment of an allocation sequence. | Low risk of bias, the outcome is not likely to be influenced due to lack of blinding. | Low risk of bias, the outcome is not likely to be influenced due to lack of blinding | No judgment possible. Unclear risk of bias. | Missing data have been imputed using appropriate methods. Low risk of bias. | Unclear risk of bias. | Unclear risk of bias, low precision due to small number of clusters. |
| *Drabo [49]* | Parallel cluster randomized trial. Randomization procedure is not described. | Allocation procedure is not described | Not described. Due to the nature of the intervention it is likely that the participants were not blinded. | Not described. Due to the nature of the intervention it is likely that the participants were not blinded. | Blinding of outcome assessment is not described. | 18% and 10% incomplete follow-up in control and intervention group. No indication that clusters were lost. | No information on selective reporting. | Lack of information on recruitment bias and baseline imbalances. Analysis did not take clustering (by DOTS-plus centers) into account and there is a possibility that the groups are not comparable on unrecorded factors. Information on the assessment of other sources of bias is not described. |
| *Authors judgment* | Unclear risk of bias. | Unclear risk of bias | Low risk of bias, the outcome is not likely to be influenced due to lack of blinding. | Low risk of bias, the outcome is not likely to be influenced due to lack of blinding. | No judgment possible. Unclear risk of bias. | High risk of bias, due to unequal distribution of patients lost and high number of patients lost. | Unclear risk of bias. | Unclear risk of bias, , low precision due to small number of clusters. |
| *Jahnavi & Sudha [57]* | Patients were randomly assigned to the food supplement group or control group. The randomization was 1:1 for the two groups. The randomization was performed by randomly shuffling envelopes that contained the study codes. | Not described if they used opaque sealed envelopes. | Not described. Due to the nature of the intervention it is likely that the participants were not blinded. | Not described. Due to the nature of the intervention it is likely that the participants were not blinded. | Not described. | 1 patient (2%) in the intervention group and 7 patients (14%) in the control group were lost. | No information on selective reporting | Information on the assessment of other sources of bias is not described. |
| *Authors judgment* | Low risk of bias, this is a sufficient randomization procedure. | Unclear risk of bias | Low risk of bias, the outcome is not likely to be influenced due to lack of blinding. | Low risk of bias, the outcome is not likely to be influenced due to lack of blinding | No judgment possible. Unclear risk of bias. | High risk of bias, due to unequal distribution of patients lost. | Unclear risk of bias. | Unclear risk of bias. |
| *Janmeja [67]* | Alternately assigned to group A or B, until each group consisted of 100 patients. | Allocation concealment was not described. | Not described. However, due to the nature of the intervention it is likely that the participants were not blinded. | The medical team was unaware of the assign of the particular patient. Blinding of psychotherapy providers was impossible | No information available | 7% of the intervention group and 47% of the control group was lost to follow-up and not retrieved. | No information on selective reporting. | Not described. |
| *Authors judgment* | High risk of bias | Unclear risk of bias | Low risk of bias, the outcome is not likely to be influenced due to lack of blinding. | Low risk of bias, the outcome is not likely to be influenced due to lack of blinding | Unclear risk of bias. | High risk of bias | Unclear risk of bias. | Unclear risk of bias |
| *Liefooghe [51]* | A systematic sampling plan was applied on a calendar basis. Alternative weeks were assigned to the intervention and control group, allocation of the first week was randomly assigned to the intervention group. | Allocation concealment was not described. | Due to the nature of the intervention it is likely that the participants were not blinded. | Blinding of the counsellors was impossible. | From treatment card. Additionally, interviewers collecting baseline information on socio-economic status and knowledge and perception on TB were blinded for which group the participants were assigned to. | No baseline data (interviews) with 22/527 (4%) patients in the intervention group and 30/551 (5%) in the control group, additionally 1 (0.2%) in intervention group and 6 (1%) in the control group were lost to follow up. | No information on selective reporting. | The reasons for the smaller effect of the intervention in patients who accept hospitalization for DOT are not clear. A first explanation is a spillover effect: as control group patients were hospitalized in the same TB wards as counselled patients, the latter could have influenced the non-counselled patients. Secondly, patients who accept hospitalization are already strongly motivated and are thus more able to compete with any problems that arise: their reasons for defaulting are probably not affected by counselling alone. |
| *Authors judgment* | High risk of bias. | Unclear risk of bias | Low risk of bias, the outcome is not likely to be influenced resulting from lack of blinding. | Low risk of bias, the outcome is not likely to be influenced resulting from lack of blinding. | Low risk of bias. | Low risk of bias, due to low loss to follow-up. | Unclear risk of bias. | High risk of bias. |
| *Lutge [62]* | Parallel cluster randomized trial in one urban and one rural district: from 209 (144 urban and 65 rural clinics, 26 (21 and 5) were eligible and 20 (16 and 4) were randomly selected. Within the two districts, the study clinics were randomly assigned in a 1:1 ratio, using a randomization list generated by the study statistician. | Clinics were allocated to intervention or control groups by the statistician using a randomization table. and no changes were made on this allocation. | No blinding was possible, due to the nature of the intervention. | No blinding was possible, due to the nature of the intervention. | Outcomes were ascertained b participating clinics using their usual procedures. | 0.2% of patients in intervention clinics and 0.7% of patients in control clinics were loss to follow-up. There was no loss of clinics. | No information on selective reporting. | Lack on information on recruitment bias and baseline imbalances. Analysis did not take clustering into account.75 (36%) Clinics was not eligible due to treatment success outside 40-70% range, 68 (33%) because annual no. of patients outside 20-150 range, 40 (19%) because no results available. Lack of control for potential confounding by HIV status and HIV treatment, this is an important limitation of this study. The co-infection rate of TB and HIV in KwaZulu-Natal is high. Additionally incentives were not provided when nurses considered patients did not need it. |
| *Authors judgment* | High risk of bias, due to the open randomization procedure. | Low risk of bias, the clinics were randomized all at once, no lack of concealment of an allocation sequence. | Low risk of bias, the outcome is not likely to be influenced by lack of blinding. | Low risk of bias, the outcome is not likely to be influenced by lack of blinding. | Unclear risk of bias, due to missing information on blinding. | Low risk of bias, due to balanced numbers across groups and no loss of clusters. | Unclear risk of bias | External validation is problematic and analysis did not adjust for clustering and HIV status. Unclear risk of bias, low precision due to small number of clusters. |
| *Martins [58]* | Parallel cluster randomized design. An independent statistician, computer generated a random allocation sequence with random varying block sizes in Stata. Allocation was stratified by community health clinic and by diagnosis of tuberculosis (smear positive and smear negative). At each study site, a research assistant randomly allocated study participants to the intervention group or control group. | The sequence was concealed from all investigators with sequentially numbered opaque sealed envelopes prepared distant from the study site. | Participants were aware of their allocation status after randomization. | Treatment providers were aware of the individual’s allocation status after randomization. | An independent observer was blinded to the intervention and determined the primary outcome (treatment completion). | Only for success data from 4 (3%) controls and 1 (0.7%) intervention was missing. For compliance, 1 (0.7%) control in intensive phase, 13 (9.7%) controls and 14 (10.2%) interventions in the continuation phase). No loss of clinics. | No information on selective reporting. | Lack on information on recruitment bias and baseline imbalances. Analysis did not take clustering into account. The control and intervention group were different in moment of attendance in the clinic, control group in the early morning, and intervention group during the mid-day. During the conflict in Dili, in the last months of the study, the treatment adherence seemed to be lower than before; although it was not significantly lower. |
| *Authors judgment* | Low risk of bias | Low risk of bias, due to blinding of concealment and randomization of the health clinics was all at once. | Low risk of bias, the outcome is not likely to be influenced by lack of blinding. | Low risk of bias, the outcome is not likely to be influenced by lack of blinding. | Low risk of bias | Low risk of bias due to balanced numbers across groups and no loss of health clinics. | Unclear risk of bias | High risk of bias due difference in time of appointment. Additionally, clustering has not been taken into account so overestimation of precision in analysis. |
| *Morisky [48]* | Patients were randomly assigned to the intervention and control group based on the last digit of their medical record number. | An open randomization procedure. | Blinding was not possible. | Blinding was not possible. | No information on blinding of outcome assessors. | Only 5 (6%) people were lost to follow up (1 (2%) intervention, 4 (9%) controls), however, this difference was not significant due to small overall numbers. | No information on selective reporting. | Not reported. |
| *Authors judgment* | High risk of bias, systematic non-random approach. | High risk of bias, selection bias could be introduced. | Low risk of bias, the outcome is not likely to be influenced by lack of blinding. | Low risk of bias, the outcome is not likely to be influenced by lack of blinding. | Unclear risk of bias. | Low risk of bias, due to low loss to follow-up. | Unclear risk of bias. | Unclear risk of bias. |
| *Sudarsanam [60]* | There was a computer generated randomization procedure. Randomization codes were generated. | Randomization codes were put in opaque sealed envelopes and opened by the dietician after dietary counselling. | Participants were not blinded. | All staff was not blinded | No information on blinding of outcome assessors. | Only 3 (5.9%) patients in the intervention and 3 (5.7%) in the control group were lost | No information on selective reporting. | Not reported. |
| *Authors judgment* | Low risk of bias | Low risk of bias | Low risk of bias, the outcome is not likely to be influenced by lack of blinding. | Low risk of bias, the outcome is not likely to be influenced by lack of blinding. | Unclear risk of bias. | Low risk of bias, due to low loss to follow-up. | No information on selective reporting. | Unclear risk of bias. |
| *Thiam [53]* | Parallel cluster randomized trial: 24 district health centers (DHCs) were stratified in two groups according to their estimated baseline sputum smear detection rate (cut-off 60 per 100.000), to account for variation in the recruitment of patients among DHCs. Within each stratum, 8 DHCs were randomly selected from the 12. | DHCs were allocated to the intervention or control groups using blocked randomization. | Participants were not blinded. | All staff was not blinded, due to the fact they were trained in the intervention. | TB nurses at the DHCs were also responsible for collection of outcome data. And outcome assessors were not blinded. However, laboratory staff were not aware of to which group the DHCs were randomized. | No indication that clusters were lost. | No information on selective reporting. | Lack on information on recruitment bias. The intervention group had a higher baseline treatment success (4 DHCs vs. 1 DHC). And DHCs randomized to the intervention were closer to Dakar (median distance intervention: 75.5 km vs control 196.5 km). However, adjustment for both has been made in the analysis. Analysis did not take clustering into account. Only 25 of 53 DHCs were fully functional and did not have other research projects; another one refused to participate. |
| *Authors judgment* | Unclear risk of bias, randomization procedure is not described. | Low risk of bias, due to blocked randomization and the randomization of the health clinics was all at once. | Low risk of bias, the outcome is not likely to be influenced by lack of blinding. | Low risk of bias, the outcome is not likely to be influenced by lack of blinding. | High risk of bias, due to partially not blinded outcome assessors. | Low risk of bias | Unclear risk of bias. | High risk of bias, due to difference in distance to Dakar and difference in baseline treatment success. Additionally, clustering has not been taken into account so overestimation of precision in analysis. |
