# Supplementary material for: The Effects of Psycho-Emotional and Socio-Economic Support for Tuberculosis Patients on Treatment Adherence and Treatment Outcomes – A Systematic Review and Meta-Analysis
Source: PLoS One. 2016 Apr 28;11(4):e0154095. doi: 10.1371/journal.pone.0154095 (PMC4849661; doi:10.1371/journal.pone.0154095)
Supplement: S2 Table — (DOCX) [file pone.0154095.s006.docx]

# S2 Table. Risk of bias assessment – New-castle Ottawa scale for non-randomized studies

| Controlled before-and-after studies, Historically controlled studies and non-randomized controlled studies | Representative of the exposed cohort | Selection of non-exposed cohort | Ascertainment of exposure | Demonstration that outcome of interest was not present at the start of the study | Comparability of cohorts on the basis of the design or analysis | Assessment of outcome | Was follow-up long enough for outcomes to occur | Adequacy of follow-up of cohorts |
| --- | --- | --- | --- | --- | --- | --- | --- | --- |
| *Bock [20]* | Patients who missed at least 25% of DOT doses over a 4-week period. | Historical controls from the same location. | Medical charts. | The outcome might have been present at the start of the study, because the study population consisted of patients who missed at least 25% of DOT doses over a 4-week period. | Signiﬁcantly more patients had isoniazid mono-resistance in the incentive than the control group (13% vs 4%; P <0.003). | Medical charts for the control group and for the intervention group. | Till treatment stopped. | Patients with incomplete follow-up were excluded from the analysis. |
| *Authors judgment* | C. Selected group of users, no star awarded. | B. Drawn from a different source, no stars awarded. | A. Secure record. | Some of the patients were included in this program/study, because they had an outcome of interest, no star awarded. | No adjustment for differences between the groups, no stars awarded. | B. Record linkage. | A. Yes. | D. No statement, due to missing description of population lost to follow-up. |
| *Cantalice Filho [59]* | TB patients > 15 years old with confirmed TB. | Historical controls from the same location. | Medical charts. | The outcome was not present at the start of the study. | Significantly more patients had associated diseases in the control group compared to the intervention group (50 vs 27%, P 0.005) | All data was obtained from medical charts, including treatment outcome. | Till treatment stopped. | No failed follow-up, due to the outcome measures (loss to follow-up and cure). |
| *Authors judgment* | A. Truly representative. | B. Drawn from a different source. | A. Secure record. | A. Yes. | No stars awarded. | B. Record linkage. | A. Yes | A. Complete follow up – all subjects accounted for. |
| *Farmer [47]* | (Extra)pulmonary TB patients from Haiti's central plateau, mostly rural people. | A district contiguous to the intervention district. | No description. | The outcome was not present at the start of the study. | Reported differences between both groups. | Clinical, laboratory and radiographic evaluation. | Measurement at 6 months, 12 months and 18 months and no further follow-up. | 4 people of the control group were not possible to locate. |
| *Authors judgment* | A. Truly representative. | B. Drawn from a different source, no star awarded. | D. No description, no star awarded. | A. Yes. | No adjustment for confounding, no stars awarded. | A. Secure records. | A. Yes. | B. Small number (7%) with unknown outcome, unlikely that bias is introduced. |
| *Garden [61]* | Homeless TB patients | Historical controls (no DOT was provided to the controls). | Data was collected from case histories using a standard questionnaire. | Unknown, the population consisted of 109 (77%) patients that were previously treated for TB. | Controls were not treated under DOT. | All data was obtained from case histories. | Till treatment stopped. | No outcome data of 6 (1.2%) people and 1 patients disappeared (<0.01%). |
| *Authors judgment* | C. Selected group of users, no star awarded. | B. Drawn from a different source, no star awarded. | A. Secure record | No star awarded due to unknown risk of bias. | No adjustment for confounding, no stars awarded. | B. Record linkage. | A. Yes. | B. Small number (1.4%) with unknown outcome, unlikely that bias is introduced. |
| *Gelmanova [44]* | TB patients that participated in at least one intervention to improve adherence before referral to the Sputnik program. | The patients were their own controls (comparison before and after inclusion in the intervention). | No description. | Yes, partly: patients that were started on a new regimen probably had defaulted, so the outcome was present at enrollment. Also some patients that continued treatment had stopped treatment for at least 4 weeks, so some may have defaulted for at least 2 months. | Not adjusted for other determinants. | Sputum testing, culture and X-ray. | The patients spent a median of 245 days in the program. | Follow-up is not during the whole treatment period, but from introduction into the program any time during treatment. |
| *Authors judgment* | C. Selected group of users, no star awarded. | No star awarded, this was not possible considering characteristics of control group. | D. No description, no star awarded. | Some of the patients were included in this program/study, because they had an outcome of interest, no star awarded. | No adjustment for confounding, no stars awarded. | A. Secure records . | A. Yes, till treatment stopped. | D. No star awarded. |
| *Lu [63]* | Migrant active TB cases. | The 3 control districts had the same characteristics as the 3 intervention districts, but other location. | TB management information system (surveillance data). | The outcome was not present at the start of the study. | Adjustment for year of patient registration, patient site, two-way interactions between patient site and year of registration, sex, age, job, and TB type. | Bacteriologic evidence for assessing the outcome. | Till treatment stopped. | Lost to follow-up of 10% in the intervention group and 15% in the control group. |
| *Authors judgment* | A. Truly representative cases for the migrant population. | B. Drawn from a different source, no star awarded. | A. Secure record. | A. Yes. | The treatment success rate was higher in the project district than in the non-project district at baseline, no adjustment on this, but there is adjustment trough difference in difference analysis approach. | A. Secure records. | A. Yes. | C. Follow up rate <90% and no description of those lost, no star awarded. |
| *Macq [55]* | New AFB positive TB patients from rural municipalities. | Municipalities that did not provide the two most consistent interventions. | Not clear. | The outcome was not present at the start of the study. | No confounders are taken into account. | Municipal register data cross-checked with the TB patient cards. | Till treatment stopped. | Only 2% and 3% of the patients in the intervention and control group abandoned their treatment. |
| *Authors judgment* | A. Truly representative for rural municipalities. | B. Drawn from a different source, no star awarded. | D. No description, no star awarded. | A. Yes. | Considering the above described, no stars awarded. | B. Record linkage. | A. Yes. | B. Small number, unlikely that bias is introduced. |
| *Soares [56]* | TB cases | Historical controls | The Brazilian national reporting system. | The outcome was not present at the start of the study. | No Adjustment for confounding | No information on this topic | Till treatment stopped. | 9.5% and 4.2 % of the control and intervention group had the status ‘unknown’. |
| *Authors judgment* | A. Truly representative for rural municipalities | B. Drawn form a different source, no star awarded. | A. Secure record | A. Yes | No star awarded. | D. No description, no star awarded. | A. Yes | B. Unlikely that bias is introduced as unknown included in denominator. |
| *Sripad [64]* | DR-TB [resistance to at least one FLD]. | Historical controls. | NTP database. | The outcome was not present at the start of the study. | % of MDR was 80% vs. 50% and % HIV+ was 1.5% vs. 9.3%, both of which may explain higher default rates in control group. No adjustment for confounding. | No description. | Till treatment stopped. | No information on this topic. |
| *Authors judgment* | A. Truly representative for rural municipalities. | B. Drawn from a different source, no star awarded. | A. Secure record. | A. Yes. | No stars awarded. | D. No description, no star awarded. | A. Yes | D. No statement, no star awarded. |
| *Wei [65]* | Migrant TB patients. | Controls were from another district, details not available to protect the patients identities. | All patients received a subsidy. Only the poor migrant TB patients received more financial incentives. Poverty was assessed by a standard questionnaire. | The outcome was not present at the start of the study. | No adjustment for confounding. | Routine TB registers were used. | Till treatment stopped. | No failed follow-up. |
| *Authors judgment* | A. Truly representative for rural municipalities. | B. Drawn from a different source, no star awarded. | B. Structured interview. | A. Yes. | No stars awarded. | B. Record linkage. | A. Yes. | A. Complete follow up – all subjects accounted for. |
| Zou [66] | Rural to urban migrant active TB cases. | Controls were from another district and contained differences compared to the intervention group. | All TB patients, regardless of economic status received a living subsidy of US$ 114 (US$ 19 per month over 6 months) and a transportation incentive of 4.4 US$. Only the poor patients received more financial incentives (was assessed by a standard questionnaire). | The outcome was not present at the start of the study. | No adjustment for confounding while significant differences in characteristics were in place. | Routine TB registers were used for data collection. | Till treatment stopped. | No failed follow-up. |
| Authors judgment | A. Truly representative for rural municipalities. | B. Drawn from a different source, no star awarded. | B. Structured interview. | A. Yes. | No stars awarded. | B. Record linkage. | A. Yes. | A. Complete follow up – all subjects accounted for. |
