# Supplementary material for: The Effects of Psycho-Emotional and Socio-Economic Support for Tuberculosis Patients on Treatment Adherence and Treatment Outcomes – A Systematic Review and Meta-Analysis
Source: PLoS One. 2016 Apr 28;11(4):e0154095. doi: 10.1371/journal.pone.0154095 (PMC4849661; doi:10.1371/journal.pone.0154095)
Supplement: S3 Table — (DOCX) [file pone.0154095.s007.docx]

# S3 Table. Risk of bias assessment – New-castle Ottawa scale for case-control studies

| Case control studies | Is the case definition adequate | Representativeness of the cases | Selection of controls | Definition of controls | Comparability of cases and controls on the basis of the design or analysis | Ascertainment of exposure | Same method for ascertainment for cases and controls | Non-response rate |
| --- | --- | --- | --- | --- | --- | --- | --- | --- |
| *Davidson [50]* | Yes, from surveillance system of the New York state. | All cases enrolled between October 1992 and March 1996. | Controls from the same population. | Not mentioned. | Adjustment for age, sex, skin color, ethnicity, resistance, psychiatric illness, living with other percent of months on enhanced incentives. | Interviews, clinical data and attendance records. | This is unclear. | Not described. |
| *Authors judgment* | B. Record linkage, no star awarded. | A. Obviously representative series of cases. | A. Community controls. | B. No description of history of adherence or non-adherence in case of retreatment, no star awarded. | Adjustment for several determinants. | C. Interviewers not blinded to case/control status, no star awarded. | B. No, no star awarded. | B. Non respondents described, no star awarded. |
| *Finlay [52]* | Yes, interviewers reviewed medical records. | Any new or re-treatment patient with pulmonary or extra pulmonary TB enrolled between January and December 2002. | Controls were from the same sample selection. | Not mentioned. | Adjustment for patient category. | The interviewers were blinded while taken and structured questionnaire. | This is unclear. | Only 26% and 43% of cases and controls were tracked and interviewed. Basic characteristics are described. |
| *Authors judgment* | A. With independent validation. | A. Obviously representative series of cases. | A. Community controls. | B. No description of source, no star awarded. | Adjustment for one additional determinant. | B. Structured interview where blind to case/control status. | B. No, no star awarded | B. Non-respondents described, however, unequal. No star awarded. |
| *Jakubowiak [45]* | Yes, based on medical records. | New pulmonary TB patients enrolled in the Tuberculosis register March-Sept 2003. | Controls from the same population. | Cases and controls are new TB patients. | Adjustment for age, sex, region, residence type and AFB smear. | Two standard questionnaires were completed, no information on blinding. | Yes the same method was used for both groups. | 84% of cases and 57% of controls were interviewed. Non-respondents are described, but no specific information is given. |
| *Authors judgment* | A. With independent validation. | A. Obviously representative series of cases. | A. Community controls. | Both groups have no history of outcome. | Multiple confounders are taken into account. | C. Interview not blinded to case/control status, no star awarded. | A. Yes. | B. Non respondents described, however they were unequal. No star awarded. |
