# Supplementary material for: The Effects of Psycho-Emotional and Socio-Economic Support for Tuberculosis Patients on Treatment Adherence and Treatment Outcomes – A Systematic Review and Meta-Analysis
Source: PLoS One. 2016 Apr 28;11(4):e0154095. doi: 10.1371/journal.pone.0154095 (PMC4849661; doi:10.1371/journal.pone.0154095)
Supplement: S1 Web Annex — (DOCX) [file pone.0154095.s008.docx]

# S1 Web Annex. Full text search strategy per database

#### Medline/Pubmed

(Tuberculosis*[TIAB]) AND (patient*[TIAB] OR treat*[TIAB]) AND (support*[TIAB] OR intervention*[TIAB] OR program*[TIAB]) AND (incentive*[TIAB] OR consult*[TIAB] OR counsel*[TIAB] OR financ*[TIAB] OR monetary[TIAB] OR socio*[TIAB] OR social*[TIAB] OR psycho*[TIAB] OR economic*[TIAB] OR transport*[TIAB] OR employ*[TIAB] OR food*[TIAB] OR nutrition*[TIAB] OR sputnik[TIAB] OR enable* OR cash transfer*[TIAB]) AND ((adherence[TIAB] OR complian*[TIAB] OR success*[TIAB] OR cure[TIAB] OR curable[TIAB] OR complet*[TIAB] OR drop*[TIAB] OR "loss to follow-up"[TIAB] OR “lost to follow-up”[TIAB] OR default*[TIAB] OR conversion[TIAB] OR converted[TIAB]) OR (financ*[TIAB] OR econom*[TIAB] OR poverty*[TIAB] OR cost*[TIAB] OR income*[TIAB] OR expenditur*[TIAB])) AND Filters: Publication date from 1990/01/01 to 2014/03/01; English; Dutch; German; French; Spanish; Portuguese; Russian

#### Embase

Tuberculosis* (ab,ti) AND (patient* or treat*) (ab,ti) AND (support* or intervention* or program*) (ab,ti) AND (incentive* or consult* or counsel* or financ* or monetary or socio* or social* or psycho* or economic* or transport* or employ* or food$ or nutrition* or sputnik or enable* or cash transfer*) (ab,ti) AND ((adherence or complian* or success* or cure or curable or complet* or drop* or 'loss to follow-up' or 'lost to follow-up' or default* or conversion or converted) (ab,ti) OR (finance* or economy* or poverty* or cost* or income* or expenditure*) (ab,ti)) AND (limit to(embase and (dutch or english or french or german or portuguese or russian or spanish) and yr="1990 - 2015"))

#### Union world conferences on Lung Health Databases

The abstract books were searched manually using the above described strategy.
